# Supplementary material for: A national audit of facilities, human and material resources for the comprehensive management of diabetes in Ghana-A 2023 update
Source: PLoS One. 2024 May 20;19(5):e0303624. doi: 10.1371/journal.pone.0303624 (PMC11104593; doi:10.1371/journal.pone.0303624)
Supplement: S1 File — (PDF) [file pone.0303624.s001.pdf]

| Type of Hea | What regio | Kindly indic | Availability | Trained Dia | If yes , how | Trained dia | If yes, how | Diabetes ec |
|---|---|---|---|---|---|---|---|---|
| Teaching Hospital | | | no | no | | yes | | yes |
| Teaching Hospital | | | yes | yes | 4 | yes | 2 | no |
| Primary care department of a teach | | | no | yes | 2 | yes | 3 | no |
| Private Hospital | | | yes | yes | one | yes | 4 | yes |
| Private Hospital | | | yes | yes | one | yes | 4 | yes |
| Teaching H | Greater Acc | AYAWASO V | yes | yes | 1 | no | | no |
| District Hos | Central Reg | Asikuma-O | yes | yes | 1 | no | | yes |
| Private hos | Greater Acc | Ayawaso ce | yes | yes | 3 | no | | no |
| Teaching H | Greater Acc | 37 Military | no | yes | 8 | yes | 4 | yes |
| Private | Greater Acc | Ayawaso w | yes | yes | 1 | yes | 1 | yes |
| District Hos | Greater Acc | La Nkwanta | yes | yes | 2 | no | | no |
| Teaching H | Ashanti Reg | Kumasi | yes | yes | 7 | yes | 8 | yes |
| District Hos | Ahafo Regio | Tano South | yes | no | | no | | no |
| District Hos | Bono Regio | Dormaa Eas | no | no | | no | | no |
| District Hos | Bono Regio | Jaman nort | no | no | | no | | no |
| District Hos | Bono Regio | Dormaa We | no | no | | no | | no |
| District Hos | Ashanti Reg | Ahafo Ano | yes | no | | yes | 2 | yes |
| Municipal H | Ahafo Regio | Asonafo No | yes | no | | no | | yes |
| District Hos | Ahafo Regio | Asunafo So | yes | no | | no | | yes |
| Municipal H | Bono Regio | Sunyani mu | no | no | | yes | 2 | no |
| Regional H | Bono Regio | Regional | no | no | | yes | 2 | no |
| Municipal H | Ashanti Reg | Tafo munici | yes | no | | no | | yes |
| District Hos | Ashanti Reg | MANHYIA S | yes | yes | 4 | yes | 2 | yes |
| Municipal H | Ashanti Reg | Kumasi Me | yes | no | | no | | yes |
| Municipal H | Ashanti Reg | Kumasi Me | yes | no | | no | | yes |
| District Hos | Bono Regio | Tain Distric | no | no | | yes | 4 | no |
| Municipal H | Ashanti Reg | Mampong | yes | no | | no | | yes |
| Municipal H | Bono East F | Atebubu M | no | no | | no | | no |
| District Hos | Bono Regio | Sene West | no | yes | 3 | no | | no |
| District Hos | Bono East F | Kintampo S | no | no | | no | | no |
| Municipal H | Central Reg | Agona Swe | no | no | | no | | no |
| District Hos | Central Reg | Abura Dunk | yes | no | | yes | 1 | yes |
| Municipal H | West North | Enchi Muni | yes | no | | yes | 3 | yes |
| District Hos | West North | Bia west | yes | no | | no | | no |
| Municipal H | West North | Bibiani anw | yes | no | | yes | 2 | yes |
| District Hos | Western Re | Effia Kwesir | yes | no | | yes | 6 | yes |
| Municipal H | Volta Regio | HOHOE MU | yes | no | | yes | 1 | yes |
| Municipal H | Western Re | JOMORO M | yes | no | | no | | no |
| Municipal H | Volta Regio | KETU SOUT | yes | no | | no | | yes |
| District Hos | Upper Wes | WA WEST | yes | no | | no | | no |
| District Hos | Upper Wes | SISALA WES | yes | no | | no | | no |
| Municipal H | Upper Wes | NANDOM | yes | no | | no | | no |
| District Hos | Central Reg | Ajumako Er | no | no | | no | | no |
| Municipal H | Bono East F | Kintampo N | no | no | | no | | no |
| Municipal H | Central Reg | Effutu distr | no | no | | no | | no |

| | | | | | | | | |
|---|---|---|---|---|---|---|---|---|
| District Hos | Central Reg | Mfantemar | yes | no | | yes | 1 | yes |
| District Hos | Central Reg | Twifo Atimo | no | no | | no | | no |
| Municipal H | Eastern Reg | Upper denk | no | no | | no | | no |
| District Hos | Eastern Reg | Abuakwa So | no | no | | no | | no |
| District Hos | Eastern Reg | Abuakwa N | no | no | | yes | 1 | yes |
| District Hos | Eastern Reg | Atiwa East | no | no | | no | | no |
| District Hos | Eastern Reg | Fanteakwa | no | no | | no | | no |
| District Hos | Eastern Reg | Birim North | no | no | | no | | no |
| District Hos | Eastern Reg | Birim Centr | no | yes | 1 | no | | no |
| District Hos | Eastern Reg | Akuapem N | yes | no | | yes | 1 | yes |
| District Hos | Eastern Reg | Nsawam Ac | no | yes | 1 | yes | 2 | no |
| Municipal H | Upper West | sisala east r | yes | no | | no | | no |
| District Hos | Upper West | Nadowli Ka | yes | no | | no | | yes |
| District Hos | Eastern Reg | Accra Metro | no | no | | yes | 4 | yes |
| District Hos | Eastern Reg | West Akim | no | no | | no | | no |
| District Hos | Eastern Reg | Ada East | no | no | | no | | no |
| District Hos | Oti Region | Biakoye | no | no | | no | | no |
| District Hos | Northern R | karaga | no | no | | no | | no |
| District Hos | Greater Acc | Ayawaso No | yes | no | | yes | 1 | no |
| District Hos | Eastern Reg | Suhum | no | no | | no | | no |
| District Hos | Eastern Reg | Upper Man | no | no | | no | | no |
| Municipal H | Greater Acc | Weija Gbaw | no | no | | no | | no |
| District Hos | North East | West Mamp | no | no | | no | | no |
| General Ho | Greater Acc | Tema Metro | no | yes | 3 | yes | 3 | yes |
| Regional Ho | Eastern Reg | New Juaben | no | no | | yes | 3 | yes |
| General hos | Greater Acc | La dade kot | no | no | | no | | no |
| District Hos | Northern R | Zabzugu | no | no | | no | | no |
| District Hos | Volta Regio | SOUTH DAY | yes | no | | no | | no |
| District Hos | Volta Regio | KETA MUNI | yes | no | | no | | yes |
| Teaching Ho | Greater Acc | Korlebu/ M | yes | yes | 3 | yes | 4 | yes |
| Teaching Ho | Greater Acc | Acca | yes | yes | 8 | yes | 2 | no |
| District Hos | Upper East | TALENSI DIS | no | no | | no | | no |
| District Hos | Upper West | LAMBUSSIE | no | no | | no | | no |
| Regional Ho | Greater Acc | Accra | yes | yes | 5 | no | | no |
| District Hos | Eastern Reg | Kwahu Sout | no | no | | no | | no |
| Municipal H | Greater Acc | Korley klott | yes | yes | 4 | no | | yes |
| District Hos | Greater Acc | Ledzokuku | yes | yes | One | no | | yes |
| District Hos | Greater Acc | Tema Metro | yes | yes | 2 | yes | 4 | yes |
| Municipal H | Greater Acc | Ledzokuku | no | yes | 5 | yes | 3 | yes |
| District Hos | Savannah R | Bole Distric | no | no | | no | | no |
| District Hos | North East | Bunkpurugu | no | no | | no | | no |
| District Hos | Upper East | Bawku Wes | no | no | | no | | no |
| Municipal H | Greater Acc | Ga East | no | no | | no | | no |
| Regional Ho | Upper East | Bolgatanga | no | yes | 1 | yes | 2 | no |
| Metropolita | Central Reg | CapeCoast | no | no | | no | | no |

| | | | | | | | | |
|---|---|---|---|---|---|---|---|---|
| specialist h | Central Reg | Effutu | no | yes | 3 | yes | 2 | no |
| District Hos | Upper East | Bongo distr | no | no | | yes | 2 | no |
| District Hos | Upper East | Builsa Nort | no | no | | no | | no |
| Municipal H | Upper East | KASENA NA | yes | no | | no | | no |
| District Hos | Savannah R | East Gonja | no | no | | no | | no |
| District Hos | Upper East | BAWKU WE | no | no | | no | | no |
| District Hos | Upper East | Bongo | no | no | | no | | no |
| District Hos | Upper East | Builsa Nort | no | no | | no | | no |
| governmen | Eastern Reg | lower Many | no | no | | no | | no |
| Central Ho | Northern R | Tamale Me | no | no | | no | | no |
| District Hos | Eastern Reg | lower many | no | no | | no | | no |
| Regional Ho | Upper Wes | WA MUNIC | yes | yes | 1 | no | | no |
| governmen | West North | Bibiani Anh | no | no | | no | | no |
| goverment | West North | Sehwi Wiav | no | no | | no | | no |
| governmen | West North | Juaboso | no | no | | no | | no |
| District Hos | Ashanti Reg | SEEKYERE S | yes | no | | no | | no |
| Municipal H | Ashanti Reg | EJISU | yes | yes | 1 | no | | no |
| District Hos | Greater Acc | Shai Osudo | yes | yes | 1 | yes | 2 | yes |
| Municipal H | Greater Acc | GA NORTH | no | no | | no | | no |
| District Hos | Upper East | Kasena Nar | no | no | | no | | no |
| District Hos | Ashanti Reg | BEKWAI | yes | no | | no | | no |
| Municipal H | Ashanti Reg | Bekwai Mu | no | no | | no | | no |
| District Hos | Ahafo Regi | Tano North | no | no | | no | | no |
| District Hos | Ahafo Regi | Asutsifi Sou | no | no | | no | | no |
| Municipal H | Ashanti Reg | BEKWAI | yes | no | | no | | no |
| District Hos | Ashanti Reg | Atwima Mp | yes | no | | no | | no |
| Municipal H | Ahafo Regi | Tano North | no | no | | no | | no |
| District Hos | Ahafo Regi | Asutsifi Nor | no | no | | no | | no |
| Polyclinic | Ahafo Regi | Asunafo No | no | no | | no | | no |
| District Hos | Ashanti Reg | Kwabre Eas | yes | no | | yes | 1 | yes |
| Municipal H | Ashanti Reg | Asante Akir | yes | no | | no | | yes |
| District Hos | Ahafo Regi | Tano North | yes | no | | no | | no |

| If yes, how | Ophthalmic | If yes, how | Psychologis | If yes, how | Opthalmolo | If yes, how | Podiatrists/ | If yes, how |
|---|---|---|---|---|---|---|---|---|
| | no | | no | | yes | | yes | |
| | yes | i'm not sure | yes | several in tl | yes | several in tl | no | |
| | yes | 4 | yes | 1 | no | | no | |
| 2 | no | | no | | no | | no | |
| 2 | no | | no | | no | | no | |
| | no | | yes | 4 | yes | 2 | no | |
| 3 | yes | 3 | yes | 1 | no | | no | |
| | yes | 3 | yes | 1 | yes | 2 | no | |
| 2 | no | | yes | 4 | yes | 6 | no | |
| 1 | no | | yes | 1 | no | | no | |
| | yes | 4 | no | | yes | 1 | no | |
| 4 | yes | 2 | yes | 5 | yes | 10 | yes | 2 |
| | yes | 2 | no | | no | | no | |
| | yes | 2 | no | | no | | no | |
| | yes | 1 | yes | 1 | yes | 1 | no | |
| | yes | 1 | no | | yes | 2 | no | |
| 1 | yes | 2 | no | | no | | no | |
| 1 | yes | 3 | no | | no | | no | |
| 1 | no | | no | | no | | no | |
| | yes | 2 | no | | no | | no | |
| | yes | 2 | no | | no | | no | |
| 2 | yes | 4 | yes | 1 | no | | no | |
| 3 | yes | 2 | no | | yes | 2 | yes | 1 |
| 4 | yes | 2 | no | | no | | yes | 1 |
| 4 | yes | 2 | no | | no | | yes | 1 |
| | yes | 1 | no | | no | | no | |
| 4 | yes | 2 | no | | no | | no | |
| | yes | 1 | no | | no | | no | |
| | yes | 1 | no | | no | | no | |
| | yes | 2 | no | | no | | no | |
| | yes | 4 | yes | 1 | no | | no | |
| 1 | yes | 2 | no | | no | | no | |
| 3 | no | | no | | no | | no | |
| | yes | 1 | no | | no | | no | |
| 2 | yes | 3 | yes | 1 | no | | no | |
| 1 | yes | 2 | no | | no | | no | |
| 1 | yes | 4 | no | | no | | no | |
| | yes | 1 | no | | no | | no | |
| 3 | yes | 2 | no | | no | | no | |
| | yes | 1 | no | | no | | no | |
| | no | | no | | no | | no | |
| | yes | 3 | no | | no | | no | |
| | yes | 2 | no | | no | | no | |
| | yes | 2 | no | | no | | no | |
| | yes | 2 | no | | no | | no | |

| | | | | | | | | |
|---|---|---|---|---|---|---|---|---|
| 1 | yes | 3 | no | | yes | 1 | no | |
| | yes | 2 | no | | yes | 1 | no | |
| | yes | 2 | no | | no | | no | |
| | yes | 2 | no | | no | | no | |
| 1 | yes | 2 | no | | no | | no | |
| | yes | 1 | no | | no | | no | |
| | yes | 1 | no | | no | | no | |
| | yes | 1 | no | | no | | no | |
| | yes | 2 | no | | yes | 1 | no | |
| 1 | yes | 3 | yes | 1 | yes | 1 | no | |
| | yes | 2 | no | | yes | 1 | no | |
| | yes | 2 | no | | no | | no | |
| 1 | yes | 1 | no | | no | | no | |
| 4 | yes | 4 | no | | no | | no | |
| | yes | 2 | no | | yes | 1 | no | |
| | yes | 2 | no | | no | | no | |
| | yes | 2 | no | | no | | no | |
| | yes | 1 | no | | no | | no | |
| | yes | 1 | no | | no | | no | |
| | yes | 1 | no | | no | | no | |
| | yes | 2 | no | | no | | no | |
| | yes | 2 | no | | yes | 1 | no | |
| | no | | no | | no | | no | |
| 2 | yes | 3 | yes | 2 | yes | 1 | no | |
| 3 | yes | 2 | no | | yes | 1 | no | |
| | yes | 2 | no | | no | | no | |
| | yes | 2 | no | | no | | no | |
| | yes | 1 | no | | no | | no | |
| 1 | yes | 1 | no | | no | | no | |
| 1 | yes | 3 | yes | 1 | yes | >5 | no | |
| | yes | 2 | yes | 3 | yes | 5 | no | |
| | yes | 2 | no | | no | | no | |
| | no | | no | | no | | no | |
| | yes | 5 | yes | 2 | yes | 2 | no | |
| | yes | 2 | no | | no | | no | |
| 1 | yes | 3 | yes | 1 | yes | 2 | no | |
| One | yes | 3 | yes | One | yes | One | yes | Two |
| 1 | yes | 5 | yes | 1 | yes | 1 | no | |
| 3 | yes | 4 | yes | 1 | yes | 1 | no | |
| | yes | 3 | no | | no | | no | |
| | yes | 2 | no | | no | | no | |
| | yes | 2 | no | | no | | no | |
| | no | | no | | no | | no | |
| | yes | 3 | no | | no | | no | |
| | yes | 2 | no | | no | | no | |

| | | | | | | | | |
|---|---|---|---|---|---|---|---|---|
| | yes | 2 | yes | 1 | yes | 1 | no | |
| | yes | 3 | yes | 1 | no | | no | |
| | yes | 3 | no | | no | | no | |
| | yes | 3 | no | | no | | no | |
| | yes | 4 | no | | no | | no | |
| | yes | 4 | no | | no | | no | |
| | yes | 2 | no | | yes | 1 | no | |
| | yes | 2 | no | | no | | no | |
| | yes | 2 | no | | no | | no | |
| | yes | 3 | yes | 1 | yes | 1 | no | |
| | yes | 3 | yes | 1 | yes | 1 | no | |
| | yes | 3 | yes | 1 | no | | no | |
| | yes | 3 | no | | yes | 1 | no | |
| | yes | 2 | no | | no | | no | |
| | yes | 1 | no | | no | | no | |
| | yes | 1 | no | | no | | no | |
| | yes | 1 | no | | no | | no | |
| 1 | yes | 2 | yes | 1 | no | | no | |
| | yes | 2 | no | | no | | no | |
| | yes | 1 | no | | no | | no | |
| | yes | 2 | no | | no | | no | |
| | yes | 5 | no | | no | | no | |
| | yes | 2 | no | | no | | no | |
| | yes | 1 | no | | no | | no | |
| | yes | 5 | no | | no | | no | |
| | yes | 1 | no | | no | | no | |
| | yes | 1 | no | | no | | no | |
| | no | | no | | no | | no | |
| | no | | no | | no | | no | |
| 2 | no | | no | | no | | no | |
| 1 | yes | 2 | no | | no | | no | |
| | yes | 2 | no | | no | | no | |

| Foot surgeo | If yes, how | Dietician(s) | If yes, how | Sphygmom | If yes, how | Glucomete | If yes, how | Ophthalmo |
|---|---|---|---|---|---|---|---|---|
| no | | no | | no | | no | | no |
| yes | 1 | yes | several in h | yes | several | yes | several | yes |
| no | | yes | 1 | yes | 8 | yes | 5 | yes |
| yes | 1 | yes | 1 | yes | 12 | yes | 14 | yes |
| yes | 1 | yes | 1 | yes | 12 | yes | 14 | yes |
| no | | yes | More than | yes | Countless | yes | Countless | yes |
| no | | yes | 1 | yes | More than | yes | More than | yes |
| no | | yes | 2 | yes | 4 | yes | 2 | yes |
| no | | yes | 6 | yes | Over 50 | yes | Over 20 | yes |
| no | | yes | 1 | yes | 4 | yes | 4 | yes |
| no | | yes | 1 | yes | at least 2 in | yes | at least 1 in | yes |
| yes | 4 | yes | 8 | yes | 4 | yes | 50 | no |
| no | | no | | yes | 15 to 20 | yes | 10 | yes |
| no | | yes | 1 | yes | 1 | yes | 8 | yes |
| no | | yes | 2 | yes | 1 | yes | 8 | no |
| no | | yes | 2 | yes | 13 | yes | 7 | yes |
| no | | no | | yes | 15 | yes | 3 | yes |
| no | | yes | 2 | yes | 3 | yes | 2 | yes |
| no | | yes | 1 | yes | 10 | yes | 5 | yes |
| no | | yes | 2 | yes | 3 | yes | 4 | yes |
| no | | yes | 2 | yes | 5 | yes | 4 | yes |
| no | | yes | 2 | yes | 3 | yes | 1 | yes |
| yes | 1 | yes | 1 | yes | 3 | yes | 3 | no |
| no | | yes | 1 | yes | 2 | yes | 2 | yes |
| no | | yes | 1 | yes | 2 | yes | 1 | yes |
| no | | yes | 1 | yes | 3 | yes | 3 | yes |
| no | | yes | 1 | yes | 3 | yes | 2 | yes |
| no | | yes | 1 | yes | 3 | yes | 2 | yes |
| no | | no | | yes | 15 | yes | 6 | yes |
| no | | no | | yes | 5 | yes | 3 | yes |
| no | | yes | 1 | yes | 10 | yes | 5 | yes |
| no | | yes | 1 | yes | 5 | yes | 6 | yes |
| no | | no | | yes | 3 | yes | 3 | yes |
| no | | yes | 1 | yes | 1 | yes | 1 | yes |
| no | | yes | 2 | yes | 2 | yes | 2 | yes |
| no | | yes | 2 | yes | 3 | yes | 1 | yes |
| no | | yes | 1 | yes | 2 | yes | 8 | yes |
| no | | yes | 1 | yes | 2 | yes | 1 | yes |
| no | | no | | yes | 3 | yes | 2 | no |
| no | | no | | yes | 1 | yes | 1 | yes |
| no | | no | | yes | 2 | yes | 4 | yes |
| no | | no | | yes | 2 | yes | 1 | yes |
| no | | no | | yes | 4 | yes | 7 | yes |
| no | | yes | 1 | yes | 6 | yes | 5 | yes |
| no | | no | | yes | 8 | yes | 7 | yes |

| | | | | | | | | | |
|---|---|---|---|---|---|---|---|---|---|
| no | | no | | yes | 2 | yes | 1 | yes |
| no | | no | | yes | 2 | yes | 2 | yes |
| no | | no | | yes | 2 | yes | 1 | yes |
| no | | no | | yes | 2 | yes | 1 | yes |
| no | | yes | 1 | yes | 2 | yes | 1 | yes |
| no | | no | | yes | 2 | yes | 2 | yes |
| no | | no | | yes | 1 | no | | yes |
| no | | no | | yes | 2 | yes | 2 | yes |
| no | | yes | 2 | yes | 3 | yes | 4 | yes |
| no | | yes | 1 | yes | 2 | yes | 2 | yes |
| no | | yes | 2 | yes | 2 | yes | 2 | yes |
| no | | no | | yes | 2 | yes | 1 | yes |
| no | | no | | yes | 1 | yes | 2 | yes |
| no | | yes | 2 | yes | 2 | yes | 2 | yes |
| no | | yes | 1 | yes | 2 | yes | 3 | yes |
| no | | no | | yes | 2 | yes | 1 | yes |
| no | | no | | yes | 3 | yes | 4 | yes |
| no | | no | | yes | 2 | no | | yes |
| no | | yes | 1 | yes | 2 | yes | 3 | yes |
| no | | yes | 1 | yes | 2 | yes | 2 | yes |
| no | | yes | 2 | yes | 3 | yes | 2 | yes |
| no | | yes | 2 | yes | 3 | yes | 3 | yes |
| no | | no | | yes | 2 | no | | yes |
| no | | yes | 3 | yes | 4 | yes | 4 | yes |
| no | | yes | 2 | yes | 3 | yes | 3 | yes |
| no | | no | | yes | 4 | yes | 3 | no |
| no | | no | | yes | 2 | no | | yes |
| no | | no | | yes | 1 | yes | 1 | yes |
| no | | yes | 1 | yes | 2 | yes | 1 | no |
| yes | 2 | yes | 1 | yes | 3 | yes | 2 | yes |
| yes | 2 | yes | 4 | yes | 4 | yes | 5 | yes |
| no | | no | | yes | 1 | yes | 1 | yes |
| no | | no | | yes | 2 | yes | 3 | no |
| no | | yes | 3 | yes | 5 | yes | 2 | no |
| no | | no | | yes | 2 | yes | 2 | yes |
| no | | yes | 2 | yes | 2 | yes | 2 | no |
| no | | yes | One | yes | Two | yes | One | yes |
| yes | 3 | yes | 1 | yes | 5 | yes | 10 | yes |
| no | | yes | 2 | yes | 4 | yes | 5 | yes |
| no | | no | | yes | 3 | yes | 3 | yes |
| no | | no | | yes | 2 | yes | 1 | yes |
| no | | no | | yes | 5 | yes | 6 | yes |
| no | | no | | yes | 4 | yes | 4 | yes |
| no | | yes | 1dietician a | yes | 4 | yes | 5 | yes |
| no | | yes | 1 | yes | 4 | yes | 3 | yes |

| | | | | | | | | |
|---|---|---|---|---|---|---|---|---|
| no | | yes | 2 | yes | 5 | yes | 4 | yes |
| no | | no | | yes | 6 | yes | 10 | yes |
| no | | no | | yes | 1 | no | | yes |
| no | | no | | yes | 7 | yes | 4 | yes |
| no | | no | | yes | 4 | yes | 4 | yes |
| no | | no | | yes | 15 | yes | 10 | yes |
| no | | no | | yes | 2 | yes | 2 | yes |
| no | | no | | yes | 2 | yes | 2 | yes |
| no | | no | | yes | 3 | yes | 2 | yes |
| no | | no | | yes | 5 | yes | 4 | yes |
| no | | yes | 1 | yes | 5 | yes | 4 | yes |
| no | | yes | 2 | yes | 2 | yes | 5 | yes |
| no | | yes | 2 | yes | 4 | yes | 5 | yes |
| no | | no | | yes | 2 | yes | 1 | no |
| no | | yes | 1 | yes | 2 | yes | 4 | yes |
| no | | no | | yes | 3 | yes | 1 | yes |
| no | | no | | yes | 2 | no | | no |
| no | | yes | 3 | yes | 6 | yes | 1 | yes |
| no | | no | | yes | 1 | yes | 1 | yes |
| no | | no | | yes | 3 | yes | 3 | yes |
| no | | no | | yes | 2 | yes | 1 | yes |
| yes | 1 general su | yes | 3 | yes | more than | yes | more than | yes |
| no | | no | | yes | 3 | yes | 3 | yes |
| no | | no | | yes | 4 | yes | 3 | yes |
| no | | no | | yes | 3 | yes | 3 | yes |
| no | | no | | yes | 2 | yes | 2 | yes |
| no | | yes | 1 | yes | 2 | yes | 2 | yes |
| no | | no | | yes | 4 | yes | 5 | yes |
| no | | no | | yes | 6 | yes | 2 | no |
| no | | yes | 3 | yes | 5 | yes | 3 | no |
| no | | no | | yes | 1 | yes | 1 | yes |
| no | | no | | yes | 3 | yes | 4 | yes |

| If yes, how | Monofilam | If yes, how | Biothensior | If yes, how | Weighing s | If yes, how | Do you hav | Do you hav |
|---|---|---|---|---|---|---|---|---|
| | yes | | yes | | no | | yes | no |
| several in tl | no | | no | | yes | several 2 fu | yes | no |
| 7 | yes | 5 | no | | yes | 4 | yes | no |
| 2 | yes | 2 | no | | yes | 4 | yes | yes |
| 2 | yes | 2 | no | | yes | 4 | yes | no |
| Countless | yes | 5 | no | | yes | 10 plus | yes | yes |
| 3 | no | | no | | yes | >5 | yes | no |
| 3 | yes | 10 | no | | yes | 2 | yes | no |
| Over 20 | no | | yes | 2 | yes | 20 | yes | yes |
| 2 | yes | Several, > 5 | no | | yes | 2 | yes | no |
| at least 2 w | no | | no | | yes | 1in DM clin | yes | no |
| | yes | 20 | no | | yes | 2 | yes | yes |
| 3 | no | | no | | yes | 10 | yes | yes |
| 1 | no | | no | | yes | 3 | yes | yes |
| | no | | no | | yes | 8 | yes | yes |
| 2 | no | | no | | yes | 8 | yes | yes |
| 1 | no | | no | | yes | 5 | yes | yes |
| 3 | no | | no | | yes | 4 | yes | yes |
| 1 | no | | no | | yes | 5 | no | yes |
| 2 | no | | no | | yes | 2 | yes | yes |
| 1 | no | | no | | yes | 4 | yes | yes |
| 1 | no | | no | | yes | 1 | yes | no |
| | no | | no | | yes | 2 | yes | yes |
| 2 | no | | no | | yes | 1 | yes | yes |
| 2 | no | | no | | yes | 1 | yes | yes |
| 2 | no | | no | | yes | 3 | yes | yes |
| 1 | no | | no | | yes | 2 | yes | yes |
| 1 | no | | no | | yes | 2 | yes | yes |
| 1 | no | | no | | yes | 5 | yes | yes |
| 1 | no | | no | | yes | 3 | yes | yes |
| 1 | no | | no | | yes | 5 | yes | yes |
| 4 | yes | 1 | no | | yes | 5 | yes | yes |
| 1 | yes | 5 | no | | yes | 1 | yes | no |
| 1 | no | | no | | yes | 1 | no | yes |
| 2 | no | | no | | yes | 1 | yes | yes |
| 1 | no | | no | | yes | 1 | yes | no |
| 5 | no | | no | | yes | 4 | yes | yes |
| 1 | no | | no | | yes | 1 | yes | yes |
| | no | | no | | yes | 1 | yes | yes |
| 1 | no | | no | | yes | 1 | yes | no |
| 1 | no | | no | | yes | 5 | no | yes |
| 1 | no | | no | | yes | 2 | yes | no |
| 1 | no | | no | | yes | 5 | yes | yes |
| 1 | yes | 1 | no | | yes | 5 | yes | yes |
| 2 | no | | no | | yes | 5 | yes | yes |

| | | | | | | | | |
|---|---|---|---|---|---|---|---|---|
| 1 | no | | no | | yes | 1 | yes | yes |
| 3 | no | | no | | yes | 3 | yes | yes |
| 1 | no | | no | | yes | 2 | yes | yes |
| 1 | no | | no | | yes | 2 | yes | yes |
| 1 | no | | no | | yes | 2 | yes | yes |
| 1 | no | | no | | yes | 2 | yes | yes |
| 1 | no | | no | | yes | 1 | yes | yes |
| 1 | no | | no | | yes | 1 | yes | yes |
| 1 | no | | no | | yes | 3 | yes | yes |
| 3 | no | | no | | yes | 1 | yes | yes |
| 1 | no | | no | | yes | 2 | yes | yes |
| 5 | no | | no | | yes | 2 | yes | no |
| 1 | no | | no | | yes | 1 | yes | yes |
| 1 | no | | no | | yes | 2 | yes | yes |
| 1 | no | | no | | yes | 1 | yes | yes |
| 1 | no | | no | | yes | 2 | yes | yes |
| 1 | no | | no | | yes | 3 | yes | yes |
| 1 | no | | no | | yes | 2 | no | no |
| 1 | no | | no | | yes | 2 | yes | yes |
| 1 | no | | no | | yes | 2 | yes | yes |
| 1 | no | | no | | no | | yes | yes |
| 1 | no | | no | | yes | 2 | yes | yes |
| 1 | no | | no | | yes | 2 | no | no |
| 2 | no | | no | | yes | 4 | yes | yes |
| 1 | no | | no | | yes | 2 | yes | yes |
| | no | | no | | yes | 2 | yes | yes |
| 1 | no | | no | | yes | 2 | yes | yes |
| 1 | no | | no | | yes | 1 | yes | yes |
| | no | | no | | yes | 1 | yes | yes |
| 2 | yes | >6 | no | | yes | 1 | yes | no |
| 3 | yes | 8 | no | | yes | 3 | yes | yes |
| 2 | no | | no | | yes | 1 | yes | yes |
| | no | | no | | yes | 3 | no | yes |
| | yes | 10 | no | | yes | 1 | yes | yes |
| 1 | no | | no | | yes | 2 | yes | yes |
| | yes | 1 | no | | yes | 1 | yes | no |
| Not sure | yes | Two | no | | yes | One | yes | no |
| 4 | yes | 1 | no | | yes | 3 | yes | no |
| 2 | no | | no | | yes | 5 | yes | yes |
| 1 | no | | no | | yes | 3 | yes | yes |
| 1 | no | | no | | yes | 2 | yes | no |
| 1 | no | | no | | yes | 6 | yes | yes |
| 1 | no | | no | | yes | 3 | yes | yes |
| 1 | no | | no | | yes | 3 | yes | yes |
| 1 | no | | no | | yes | 4 | yes | yes |

| | | | | | | | | |
|---|---|---|---|---|---|---|---|---|
| 2 | no | | no | | yes | 3 | yes | yes |
| 3 | no | | no | | yes | 10 | yes | yes |
| 2 | no | | no | | yes | 1 | yes | no |
| 4 | no | | no | | yes | 1 | yes | yes |
| 1 | no | | no | | yes | 3 | no | no |
| 2 | no | | no | | yes | 4 | yes | yes |
| 1 | no | | no | | yes | 3 | yes | yes |
| 1 | no | | no | | yes | 2 | yes | yes |
| 1 | no | | no | | yes | 3 | yes | yes |
| 1 | no | | no | | yes | 4 | yes | yes |
| 1 | no | | no | | yes | 3 | yes | yes |
| 1 | no | | no | | yes | 2 | yes | yes |
| 1 | no | | no | | yes | 4 | yes | yes |
| | no | | no | | yes | 2 | no | no |
| 1 | no | | no | | yes | 3 | yes | yes |
| 1 | no | | no | | yes | 2 | yes | yes |
| | no | | no | | yes | 1 | yes | yes |
| Per consult | no | | no | | yes | 2 | yes | no |
| 1 | no | | no | | yes | 1 | yes | yes |
| 1 | no | | no | | yes | 2 | yes | no |
| 1 | no | | no | | yes | 1 | no | yes |
| 5 | no | | no | | yes | more 5 | yes | yes |
| 1 | no | | no | | yes | 4 | yes | yes |
| 1 | no | | no | | yes | 2 | yes | yes |
| 1 | no | | no | | yes | 2 | yes | no |
| 1 | no | | no | | yes | 1 | yes | no |
| 1 | no | | no | | yes | 2 | yes | yes |
| 1 | no | | no | | yes | 5 | yes | yes |
| | no | | no | | yes | 2 | yes | yes |
| | no | | no | | yes | 3 | yes | yes |
| 1 | no | | no | | yes | 1 | yes | yes |
| 1 | no | | no | | yes | 4 | yes | yes |

| Do you hav | Do you hav | Do you hav | Do you hav | Do you hav | Do you hav | Do you hav | Do you hav | Availability |
|---|---|---|---|---|---|---|---|---|
| yes | yes | yes | yes | yes | no | no | no | no |
| yes | yes | yes | yes | yes | no | no | yes | yes |
| yes | no | yes | yes | yes | no | no | yes | yes |
| yes | no | yes | yes | yes | no | no | yes | yes |
| yes | no | yes | yes | yes | no | no | yes | yes |
| yes | yes | yes | yes | yes | yes | yes | yes | yes |
| yes | no | yes | yes | yes | no | no | yes | yes |
| yes | yes | yes | yes | yes | yes | yes | yes | yes |
| yes | yes | yes | yes | yes | no | yes | yes | yes |
| yes | yes | yes | yes | yes | no | no | yes | yes |
| yes | no | yes | yes | yes | no | no | yes | yes |
| yes | yes | yes | yes | yes | yes | yes | yes | yes |
| yes | yes | no | no | no | no | no | no | yes |
| yes | no | no | no | no | no | no | no | yes |
| yes | yes | no | no | no | no | no | no | yes |
| yes | yes | no | no | no | no | no | no | yes |
| yes | no | yes | no | no | no | no | no | yes |
| yes | no | yes | yes | yes | no | no | yes | yes |
| yes | yes | no | yes | no | no | no | no | yes |
| yes | yes | no | no | no | no | no | yes | yes |
| yes | no | no | yes | yes | no | no | yes | yes |
| yes | no | yes | yes | yes | no | no | yes | yes |
| yes | no | yes | yes | yes | no | no | yes | yes |
| yes | yes | yes | yes | yes | no | no | yes | yes |
| yes | yes | yes | yes | yes | no | no | yes | yes |
| yes | yes | yes | yes | yes | yes | yes | yes | yes |
| yes | yes | no | yes | yes | no | no | yes | yes |
| yes | yes | yes | no | no | no | no | no | no |
| yes | yes | no | no | no | no | no | no | yes |
| yes | yes | yes | yes | yes | yes | yes | yes | yes |
| yes | yes | yes | yes | yes | yes | yes | no | yes |
| yes | no | no | no | no | yes | no | no | yes |
| yes | no | no | yes | yes | no | no | yes | yes |
| yes | no | no | no | no | no | no | no | no |
| yes | yes | yes | yes | yes | no | no | yes | yes |
| yes | no | yes | yes | yes | no | no | yes | yes |
| yes | yes | yes | yes | yes | no | no | yes | yes |
| yes | yes | no | yes | yes | no | no | yes | yes |
| yes | yes | no | yes | yes | no | no | yes | yes |
| yes | no | no | yes | yes | no | no | yes | yes |
| yes | no | no | no | no | no | no | no | yes |
| yes | no | no | no | yes | no | no | yes | yes |
| yes | yes | yes | yes | yes | yes | yes | yes | yes |
| yes | yes | yes | yes | yes | yes | yes | yes | yes |
| yes | yes | no | no | no | no | no | no | yes |

| | | | | | | | | |
|---|---|---|---|---|---|---|---|---|
| yes | yes | yes | yes | yes | no | no | yes | yes |
| yes | yes | no | no | no | no | no | no | yes |
| yes | yes | yes | yes | yes | yes | yes | yes | yes |
| yes | yes | no | yes | yes | yes | yes | yes | yes |
| yes | yes | yes | yes | yes | yes | yes | yes | yes |
| yes | yes | yes | yes | no | no | no | no | yes |
| yes | yes | no | yes | yes | yes | yes | yes | yes |
| yes | yes | yes | yes | yes | yes | yes | yes | yes |
| yes | yes | no | yes | yes | yes | yes | yes | yes |
| yes | yes | yes | yes | yes | yes | yes | yes | yes |
| yes | yes | yes | yes | yes | yes | yes | yes | yes |
| yes | no | no | yes | yes | no | no | yes | yes |
| yes | no | yes | yes | yes | no | no | yes | yes |
| yes | yes | yes | yes | yes | yes | yes | yes | yes |
| yes | yes | no | no | no | no | no | no | yes |
| yes | yes | yes | yes | yes | yes | yes | yes | yes |
| yes | yes | yes | yes | yes | yes | yes | yes | yes |
| no | no | no | no | no | no | no | no | yes |
| yes | yes | yes | yes | yes | yes | yes | yes | yes |
| yes | yes | yes | yes | yes | yes | yes | yes | yes |
| yes | yes | no | no | no | no | no | no | yes |
| yes | yes | yes | yes | yes | yes | yes | yes | yes |
| no | no | no | no | no | no | no | no | yes |
| yes | yes | yes | yes | yes | yes | yes | yes | yes |
| yes | yes | yes | yes | yes | yes | yes | yes | yes |
| yes | yes | yes | yes | yes | yes | yes | yes | yes |
| yes | yes | yes | yes | yes | yes | yes | yes | yes |
| yes | yes | yes | yes | yes | no | no | yes | yes |
| yes | no | no | yes | yes | no | no | yes | yes |
| yes | no | yes | yes | yes | no | no | yes | yes |
| yes | yes | yes | yes | yes | yes | yes | yes | yes |
| yes | no | no | no | no | no | no | no | yes |
| yes | no | no | no | no | no | no | no | yes |
| yes | yes | yes | yes | yes | no | no | yes | yes |
| yes | yes | yes | yes | yes | yes | yes | yes | yes |
| yes | yes | yes | yes | yes | yes | yes | yes | yes |
| yes | no | no | yes | yes | no | no | yes | yes |
| yes | yes | yes | yes | yes | no | no | yes | yes |
| yes | yes | yes | yes | yes | yes | yes | yes | yes |
| yes | yes | no | yes | yes | no | yes | yes | yes |
| no | no | no | yes | yes | no | no | yes | yes |
| yes | yes | yes | yes | yes | no | no | yes | yes |
| no | no | no | yes | yes | no | no | yes | yes |
| yes | yes | yes | yes | yes | no | no | yes | yes |
| yes | yes | yes | yes | yes | yes | yes | yes | yes |

| | | | | | | | | |
|---|---|---|---|---|---|---|---|---|
| yes | yes | yes | yes | yes | yes | yes | yes | yes |
| yes | no | no | no | no | no | no | no | yes |
| yes | no | no | no | no | no | no | no | yes |
| yes | no | no | yes | yes | no | no | yes | yes |
| no | no | no | no | no | no | no | no | yes |
| yes | no | no | yes | no | no | no | yes | yes |
| yes | yes | yes | yes | yes | yes | yes | yes | yes |
| yes | no | no | yes | yes | no | no | no | yes |
| yes | yes | yes | yes | yes | no | no | yes | yes |
| yes | yes | yes | yes | yes | yes | yes | yes | yes |
| yes | yes | no | yes | yes | no | no | yes | yes |
| yes | yes | yes | yes | yes | no | yes | yes | yes |
| yes | yes | yes | yes | yes | yes | yes | yes | yes |
| no | no | no | no | no | no | no | no | yes |
| yes | yes | no | yes | yes | no | no | yes | yes |
| yes | no | yes | yes | yes | no | no | yes | yes |
| yes | yes | no | no | no | no | no | no | yes |
| yes | no | yes | yes | yes | no | no | yes | yes |
| yes | yes | no | no | no | no | no | no | yes |
| no | no | no | no | no | no | no | no | yes |
| yes | no | no | yes | yes | no | no | yes | yes |
| yes | yes | yes | yes | yes | no | no | yes | yes |
| no | no | no | no | no | no | no | no | yes |
| yes | no | yes | yes | yes | no | no | yes | yes |
| yes | no | yes | yes | yes | no | no | yes | yes |
| yes | no | no | yes | yes | no | no | yes | yes |
| yes | yes | no | no | no | no | no | no | yes |
| yes | yes | no | no | no | no | no | no | yes |
| yes | yes | no | no | no | no | no | no | yes |
| yes | yes | no | yes | yes | no | no | yes | yes |
| yes | no | no | no | no | no | no | no | yes |
| yes | yes | yes | yes | yes | yes | yes | yes | yes |

| If yes kindly | Availability | If yes, kindl | Availability | If yes kindly | Availability | If yes, kind | Availability | If yes, kind |
|---|---|---|---|---|---|---|---|---|
| | yes | Below avera | no | | yes | Below avera | no | |
| Excellent | no | | yes | Good | yes | Below avera | yes | Below avera |
| Excellent | yes | Good | yes | Excellent | yes | Good | yes | Average |
| Excellent | yes | Good | yes | Excellent | yes | Good | no | |
| Excellent | yes | Good | yes | Excellent | yes | Good | no | |
| Excellent | yes | Excellent | yes | Excellent | yes | Excellent | yes | Excellent |
| Excellent | yes | Average | yes | Good | no | | no | |
| Excellent | no | | yes | Good | yes | Average | no | |
| Good | yes | Good | yes | Good | yes | Good | yes | Good |
| Good | yes | Good | yes | Good | yes | Good | yes | Good |
| Good | no | | yes | Good | no | | no | |
| Good | yes | Excellent | yes | Good | no | | yes | Good |
| Average | no | | no | | no | | no | |
| Excellent | yes | Excellent | no | | no | | no | |
| Good | yes | Good | yes | Good | yes | Good | no | |
| Good | yes | Good | no | | yes | Good | yes | Good |
| Excellent | yes | Excellent | yes | Excellent | no | | yes | Good |
| Excellent | yes | Excellent | yes | Excellent | no | | no | |
| Good | no | | yes | Good | yes | Average | yes | Good |
| Good | yes | Good | yes | Good | no | | no | |
| Good | yes | Good | yes | Good | no | | no | |
| Good | no | | yes | Average | no | | no | |
| Good | yes | Good | yes | Good | no | | yes | Good |
| Average | no | | no | | yes | Average | no | |
| Average | no | | no | | no | | no | |
| Excellent | yes | Excellent | yes | Good | yes | Good | yes | Good |
| Excellent | yes | Good | yes | Good | yes | Excellent | yes | Excellent |
| | no | | no | | no | | no | |
| Excellent | yes | Good | yes | Good | yes | Good | yes | Good |
| Good | yes | Good | yes | Good | no | | no | |
| Average | yes | Average | yes | Average | yes | Average | yes | Average |
| Good | yes | Good | yes | Good | yes | Good | yes | Good |
| Good | no | | yes | Good | no | | no | |
| | no | | yes | Average | no | | no | |
| Excellent | no | | yes | Excellent | yes | Excellent | yes | Excellent |
| Average | no | | yes | Average | yes | Below avera | yes | Average |
| Average | yes | Average | yes | Average | no | | no | |
| Excellent | yes | Average | yes | Below avera | no | | no | |
| Average | no | | no | | no | | no | |
| Below avera | no | | no | | no | | yes | Below avera |
| Average | no | | no | | no | | no | |
| Excellent | no | | yes | Excellent | no | | yes | Excellent |
| Good | yes | Good | yes | Good | no | | yes | Good |
| Good | yes | Good | yes | Good | yes | Good | yes | Good |
| Good | yes | Good | yes | Good | no | | no | |

| | | | | | | | | |
|---|---|---|---|---|---|---|---|---|
| Average | yes | Average | yes | Good | no | | yes | Average |
| Good | no | | no | | no | | no | |
| Good | no | | yes | Good | no | | no | |
| Good | no | | yes | Good | no | | no | |
| Good | no | | yes | Good | no | | no | |
| Average | no | | yes | Average | no | | no | |
| Good | no | | yes | Good | no | | no | |
| Good | no | | yes | Good | no | | no | |
| Good | yes | Average | yes | Good | no | | no | |
| Good | yes | Good | yes | Good | no | | no | |
| Good | no | | yes | Good | no | | yes | Average |
| Below avera | no | | yes | Average | no | | no | |
| Below avera | no | | no | | no | | yes | Below avera |
| Good | yes | Good | yes | Good | no | | no | |
| Good | no | | yes | Good | no | | no | |
| Good | no | | yes | Good | no | | no | |
| Good | no | | yes | Good | no | | no | |
| Average | no | | yes | Average | no | | no | |
| Good | no | | yes | Good | no | | no | |
| Good | no | | yes | Good | no | | no | |
| Good | no | | yes | Good | no | | no | |
| Average | no | | yes | Average | no | | no | |
| Good | no | | yes | Good | no | | no | |
| Excellent | no | | yes | Excellent | yes | Good | no | |
| Good | no | | yes | Good | no | | no | |
| Good | no | | yes | Good | no | | no | |
| Average | no | | yes | Average | no | | no | |
| Excellent | no | | yes | Excellent | no | | no | |
| Excellent | no | | yes | Average | no | | no | |
| Average | yes | Good | yes | Excellent | yes | Average | yes | Average |
| Good | yes | Average | yes | Good | yes | Good | yes | Good |
| Average | no | | no | | no | | no | |
| Below avera | no | | no | | no | | no | |
| Good | yes | Good | yes | Average | no | | yes | Average |
| Good | no | | yes | Good | no | | no | |
| Good | no | | yes | Excellent | no | | no | |
| Good | no | | yes | Average | no | | no | |
| Good | yes | Average | yes | Good | yes | Below avera | yes | Average |
| Good | yes | Good | yes | Good | yes | Average | yes | Below avera |
| Good | no | | yes | Good | no | | no | |
| Good | no | | yes | Average | no | | no | |
| Good | no | | yes | Good | no | | no | |
| Good | no | | yes | Good | no | | no | |
| Good | no | | yes | Good | no | | no | |
| Good | no | | yes | Good | no | | no | |

| | | | | | | | | |
|---|---|---|---|---|---|---|---|---|
| Good | yes | Good | yes | Good | yes | Average | yes | Average |
| Average | yes | Average | yes | Average | no | | yes | Average |
| Below aver | no | | yes | Below aver | no | | no | |
| Average | no | | no | | no | | no | |
| Average | no | | yes | Average | no | | no | |
| Below aver | no | | no | | no | | no | |
| Below aver | no | | no | | no | | no | |
| Average | no | | yes | Average | no | | no | |
| Good | no | | yes | Good | no | | no | |
| Good | no | | yes | Good | no | | no | |
| Excellent | no | | yes | Excellent | no | | no | |
| Good | no | | yes | Excellent | yes | Good | yes | Good |
| Excellent | no | | yes | Good | no | | yes | Good |
| Good | no | | yes | Good | no | | no | |
| Good | no | | yes | Good | no | | no | |
| Excellent | yes | Below aver | yes | Good | yes | Below aver | yes | Below aver |
| Excellent | yes | Average | yes | Average | no | | yes | Average |
| Good | yes | Good | yes | Average | no | | no | |
| Excellent | no | | no | | no | | no | |
| Good | no | | yes | Good | no | | no | |
| Good | no | | no | | no | | no | |
| Good | yes | Good | yes | Good | yes | Good | yes | Good |
| Good | no | | yes | Good | no | | yes | Good |
| Good | no | | yes | Good | no | | no | |
| Excellent | no | | yes | Excellent | no | | no | |
| Good | no | | yes | Good | no | | no | |
| Good | no | | yes | Good | no | | no | |
| Good | no | | yes | Good | no | | no | |
| Average | no | | yes | Average | no | | no | |
| Average | no | | yes | Below aver | no | | yes | Below aver |
| Excellent | yes | Excellent | yes | Excellent | no | | no | |
| Good | yes | Good | yes | Good | no | | no | |

| Availability | If yes,  kind | Availability | If yes,  kind | Availability | If yes,  kind | Availability | If yes,  kind | Availability |
|---|---|---|---|---|---|---|---|---|
| no | | no | | no | | no | | no |
| yes | Below aver | yes | Average | yes | Good | yes | Excellent | yes |
| yes | Average | yes | Excellent | yes | Average | yes | Excellent | yes |
| yes | Excellent | no | | yes | Excellent | yes | Excellent | yes |
| yes | Excellent | no | | yes | Excellent | yes | Excellent | yes |
| yes | Excellent | yes | Excellent | yes | Good | yes | Excellent | yes |
| no | | no | | no | | yes | Excellent | yes |
| yes | Excellent | yes | Excellent | yes | Average | yes | Excellent | yes |
| yes | Good | yes | Good | yes | Good | yes | Good | yes |
| yes | Good | yes | Good | yes | Average | yes | Excellent | yes |
| no | | yes | Good | no | | yes | Excellent | yes |
| yes | Excellent | yes | Good | yes | Good | yes | Excellent | yes |
| no | | no | | no | | yes | Good | yes |
| no | | yes | Excellent | no | | yes | Excellent | yes |
| no | | yes | Good | no | | yes | Good | yes |
| no | | yes | Good | no | | yes | Good | yes |
| no | | yes | Excellent | yes | Excellent | yes | Excellent | yes |
| no | | yes | Excellent | no | | yes | Excellent | yes |
| yes | Good | yes | Average | no | | yes | Excellent | yes |
| no | | yes | Good | yes | Good | yes | Good | yes |
| no | | yes | Good | no | | yes | Good | yes |
| yes | Below aver | no | | no | | yes | Good | yes |
| no | | no | | no | | yes | Excellent | yes |
| no | | no | | no | | yes | Excellent | yes |
| no | | no | | no | | yes | Excellent | yes |
| yes | Good | yes | Good | no | | yes | Excellent | yes |
| yes | Excellent | yes | Excellent | no | | yes | Excellent | yes |
| no | | no | | no | | yes | Good | yes |
| yes | Good | yes | Good | yes | Good | yes | Excellent | yes |
| yes | Good | no | | no | | yes | Good | yes |
| yes | Average | yes | Average | no | | yes | Good | yes |
| yes | Good | yes | Good | no | | yes | Good | yes |
| no | | no | | no | | yes | Excellent | yes |
| no | | no | | no | | yes | Excellent | yes |
| yes | Excellent | yes | Excellent | no | | yes | Excellent | yes |
| no | | yes | Below aver | no | | yes | Excellent | yes |
| yes | Average | no | | no | | yes | Good | yes |
| no | | no | | no | | yes | Excellent | yes |
| no | | no | | no | | yes | Average | yes |
| no | | no | | no | | yes | Good | yes |
| no | | no | | no | | yes | Excellent | yes |
| no | | yes | Excellent | no | | yes | Excellent | yes |
| yes | Good | no | | no | | yes | Good | yes |
| yes | Good | no | | no | | yes | Good | yes |
| no | | yes | Good | no | | yes | Good | yes |

| | | | | | | | | |
|---|---|---|---|---|---|---|---|---|
| no | | no | | no | | yes | Good | yes |
| no | | no | | no | | yes | Good | yes |
| no | | no | | no | | yes | Good | yes |
| no | | no | | no | | yes | Good | yes |
| no | | no | | no | | yes | Good | yes |
| no | | no | | no | | yes | Good | yes |
| no | | no | | no | | yes | Good | yes |
| no | | no | | no | | yes | Good | yes |
| no | | no | | no | | yes | Good | yes |
| no | | no | | no | | yes | Excellent | yes |
| no | | no | | no | | yes | Good | yes |
| no | | no | | no | | yes | Good | yes |
| no | | no | | no | | yes | Good | yes |
| yes | Average | yes | Average | no | | yes | Good | yes |
| no | | no | | no | | yes | Good | yes |
| no | | no | | no | | yes | Good | yes |
| no | | no | | no | | yes | Good | yes |
| no | | no | | no | | yes | Average | yes |
| yes | Good | yes | Good | no | | yes | Good | yes |
| no | | no | | no | | yes | Good | yes |
| no | | no | | no | | yes | Good | yes |
| no | | no | | no | | yes | Average | yes |
| no | | no | | no | | yes | Good | no |
| yes | Good | yes | Good | no | | yes | Excellent | yes |
| yes | Good | yes | Good | no | | yes | Good | yes |
| no | | no | | no | | yes | Good | yes |
| no | | no | | no | | yes | Good | yes |
| no | | no | | no | | yes | Excellent | yes |
| no | | yes | Average | no | | yes | Average | yes |
| yes | Average | yes | Average | yes | Below avera | yes | Excellent | yes |
| yes | Good | yes | Good | yes | Good | yes | Good | yes |
| no | | no | | no | | yes | Good | yes |
| no | | no | | no | | yes | Good | yes |
| yes | Average | yes | Below avera | no | | yes | Good | yes |
| no | | no | | no | | yes | Good | yes |
| yes | Good | yes | Good | yes | Below avera | yes | Excellent | yes |
| no | | no | | no | | yes | Good | yes |
| yes | Good | yes | Average | yes | Below avera | yes | Excellent | yes |
| yes | Average | yes | Below avera | no | | yes | Good | yes |
| no | | no | | no | | yes | Good | yes |
| no | | no | | no | | yes | Good | yes |
| no | | no | | no | | yes | Good | yes |
| no | | no | | no | | yes | Good | yes |
| yes | Average | no | | no | | yes | Good | yes |
| yes | Good | no | | no | | yes | Good | yes |

| | | | | | | | | |
|---|---|---|---|---|---|---|---|---|
| yes | Average | no | | no | | yes | Good | yes |
| no | | no | | no | | yes | Good | yes |
| no | | no | | no | | yes | Average | yes |
| no | | no | | no | | yes | Average | no |
| no | | no | | no | | yes | Average | yes |
| no | | no | | no | | yes | Average | yes |
| no | | no | | no | | yes | Below avera | yes |
| no | | no | | no | | yes | Average | yes |
| no | | no | | no | | yes | Good | yes |
| no | | no | | no | | yes | Good | yes |
| no | | no | | no | | yes | Excellent | yes |
| no | | yes | Good | no | | yes | Excellent | yes |
| yes | Good | no | | yes | Average | yes | Excellent | yes |
| no | | no | | no | | yes | Good | yes |
| no | | no | | no | | yes | Good | yes |
| yes | Good | yes | Below avera | no | | yes | Excellent | yes |
| no | | no | | no | | yes | Excellent | yes |
| no | | no | | no | | yes | Excellent | yes |
| no | | no | | no | | yes | Excellent | yes |
| no | | no | | no | | yes | Good | yes |
| no | | yes | Average | no | | yes | Excellent | yes |
| no | | yes | Good | no | | yes | Good | yes |
| no | | no | | no | | yes | Good | yes |
| no | | no | | no | | yes | Good | yes |
| yes | Below avera | yes | Below avera | yes | Below avera | yes | Good | yes |
| no | | no | | no | | yes | Excellent | yes |
| no | | no | | no | | yes | Good | yes |
| no | | no | | no | | yes | Good | yes |
| no | | no | | no | | yes | Good | yes |
| no | | yes | Below avera | yes | Below avera | yes | Excellent | yes |
| no | | yes | Excellent | no | | yes | Excellent | yes |
| no | | no | | no | | yes | Good | yes |

| If yes, kind | Availability | If yes, kind | Availability | If yes, kind | Availability | If yes, kind | Availability | If yes, kind |
|---|---|---|---|---|---|---|---|---|
| | no | | yes | Below aver | no | | no | |
| Excellent | yes | Good | yes | Average | yes | Good | yes | Below aver |
| Excellent | yes | Good | yes | Good | yes | Good | yes | Average |
| Excellent | yes | Excellent | yes | Excellent | yes | Excellent | yes | Good |
| Excellent | yes | Excellent | yes | Excellent | yes | Excellent | yes | Average |
| Excellent | yes | Excellent | yes | Excellent | yes | Excellent | no | |
| Good | no | | no | | yes | Good | no | |
| Excellent | yes | Good | yes | Good | yes | Average | no | |
| Good | yes | Good | yes | Good | yes | Good | yes | Good |
| Excellent | yes | Excellent | yes | Good | yes | Below aver | yes | Average |
| Excellent | yes | Good | yes | Good | no | | no | |
| Excellent | yes | Excellent | yes | Excellent | yes | Excellent | yes | Good |
| Good | no | | no | | no | | no | |
| Excellent | no | | no | | yes | Excellent | no | |
| Good | no | | no | | no | | no | |
| Good | no | | no | | no | | no | |
| Excellent | no | | no | | no | | no | |
| Excellent | no | | no | | no | | no | |
| Excellent | no | | no | | no | | no | |
| Good | no | | no | | no | | no | |
| Good | no | | no | | no | | no | |
| Good | yes | Below aver | no | | yes | Average | no | |
| Excellent | no | | no | | yes | Excellent | no | |
| Excellent | no | | no | | yes | Good | no | |
| Good | no | | no | | no | | no | |
| Good | no | | no | | no | | no | |
| Excellent | no | | no | | yes | Good | no | |
| Good | no | | no | | no | | no | |
| Excellent | no | | no | | no | | no | |
| Good | no | | no | | no | | no | |
| Good | no | | no | | no | | no | |
| Good | no | | no | | no | | no | |
| Excellent | no | | no | | no | | no | |
| Excellent | no | | no | | yes | Excellent | no | |
| Excellent | yes | Below aver | no | | yes | Below aver | no | |
| Excellent | no | | no | | no | | no | |
| Average | no | | no | | no | | no | |
| Good | no | | no | | no | | no | |
| Good | no | | no | | no | | no | |
| Good | no | | no | | no | | no | |
| Below aver | no | | no | | no | | no | |
| Excellent | no | | no | | no | | no | |
| Good | no | | no | | no | | no | |
| Good | no | | no | | no | | no | |
| Good | no | | no | | no | | no | |

| | | | | | | | | |
|---|---|---|---|---|---|---|---|---|
| Good | no | | no | | no | | no | |
| Good | no | | no | | no | | no | |
| Good | no | | no | | no | | no | |
| Good | no | | no | | no | | no | |
| Good | no | | no | | no | | no | |
| Good | no | | no | | no | | no | |
| Good | no | | no | | no | | no | |
| Good | no | | no | | no | | no | |
| Good | no | | no | | no | | no | |
| Good | no | | no | | yes | Average | no | |
| Good | no | | no | | no | | no | |
| Good | no | | no | | no | | no | |
| Good | no | | no | | no | | no | |
| Good | no | | no | | no | | no | |
| Good | no | | no | | no | | no | |
| Good | no | | no | | no | | no | |
| Good | no | | no | | no | | no | |
| Average | no | | no | | no | | no | |
| Good | no | | no | | no | | no | |
| Good | no | | no | | no | | no | |
| Good | no | | no | | no | | no | |
| Average | no | | no | | no | | no | |
| | no | | no | | no | | no | |
| Excellent | yes | Good | no | | yes | Good | no | |
| Good | yes | Good | yes | Good | yes | Good | no | |
| Good | no | | no | | no | | no | |
| Good | no | | no | | no | | no | |
| Excellent | no | | no | | no | | no | |
| Average | no | | no | | yes | Average | no | |
| Excellent | yes | Good | yes | Below aver | yes | Excellent | yes | Below aver |
| Good | yes | Good | yes | Good | yes | Good | yes | Good |
| Good | no | | no | | no | | no | |
| Good | no | | no | | no | | no | |
| Good | yes | Average | no | | yes | Good | no | |
| Good | no | | no | | no | | no | |
| Excellent | yes | Average | yes | Good | yes | Average | yes | Below aver |
| Good | no | | no | | no | | no | |
| Excellent | yes | Average | yes | Below aver | yes | Excellent | yes | Below aver |
| Good | yes | Average | yes | Average | yes | Below aver | no | |
| Good | no | | no | | no | | no | |
| Good | no | | no | | no | | no | |
| Good | no | | no | | no | | no | |
| Good | no | | no | | no | | no | |
| Good | no | | no | | no | | no | |
| Good | no | | no | | no | | no | |

| | | | | | | | | |
|---|---|---|---|---|---|---|---|---|
| Good | yes | Average | no | | no | | no | |
| Good | yes | Average | no | | no | | no | |
| Average | no | | no | | no | | no | |
| | yes | Average | no | | no | | no | |
| Average | no | | no | | no | | no | |
| Average | no | | no | | no | | no | |
| Below aver | no | | no | | no | | no | |
| Average | no | | no | | no | | no | |
| Good | no | | no | | no | | no | |
| Good | no | | no | | no | | no | |
| Excellent | no | | no | | no | | no | |
| Excellent | yes | Good | yes | Good | yes | Good | no | |
| Excellent | yes | Good | no | | yes | Average | no | |
| Good | no | | no | | no | | no | |
| Good | no | | no | | no | | no | |
| Excellent | no | | yes | Below aver | yes | Average | no | |
| Excellent | no | | no | | no | | no | |
| Excellent | no | | no | | yes | Average | no | |
| Excellent | no | | no | | yes | Average | no | |
| Good | no | | no | | no | | no | |
| Excellent | no | | no | | yes | Below aver | no | |
| Good | no | | yes | Good | yes | Good | no | |
| Good | no | | no | | no | | no | |
| Good | no | | no | | no | | no | |
| Excellent | yes | Average | yes | Average | yes | Good | yes | Below aver |
| Excellent | no | | no | | yes | Good | no | |
| Good | no | | no | | no | | no | |
| Good | no | | no | | no | | no | |
| Good | no | | no | | no | | no | |
| Good | yes | Below aver | no | | yes | Below aver | no | |
| Excellent | yes | Below aver | no | | yes | Excellent | no | |
| Good | no | | no | | no | | no | |

| Other medi | If yes, kind | Other medi | If yes, kind | Other medi | If yes, kind | Payment Sc | Payment Sc | Payment Sc |
|---|---|---|---|---|---|---|---|---|
| Hh | Below average | | Below average | | Below aver | yes | yes | no |
| nil | | | | | | yes | yes | yes |
| | | | | | | yes | yes | yes |
| Neuropathi | Excellent | | | | | no | yes | yes |
| | | | | | | no | yes | yes |
| | | | | | | no | yes | yes |
| | | | | | | yes | no | yes |
| | | | | | | no | yes | yes |
| | | | | | | yes | yes | yes |
| BP medications, lipid lo | | None | | | | no | yes | no |
| metformin | Excellent | | | | | yes | yes | yes |
| none | | | | | | yes | yes | yes |
| | | | | | | yes | yes | yes |
| none | | non | | none | | yes | yes | yes |
| none | | none | | none | | yes | yes | yes |
| none | | none | | none | | yes | no | yes |
| | | | | | | yes | no | yes |
| | | | | | | yes | yes | yes |
| | | | | | | yes | no | yes |
| None | | None | | None | | yes | no | no |
| None | | None | | None | | yes | no | no |
| | | | | | | yes | yes | yes |
| | Good | Statins | Good | | | yes | no | yes |
| | | | | | | yes | no | yes |
| none | | | | | | yes | no | yes |
| None | | None | | None | | yes | no | yes |
| | | | | | | yes | yes | yes |
| none | | none | | none | | yes | no | no |
| none | | none | | none | | yes | no | yes |
| none | | none | | none | | yes | no | no |
| none | | none | | none | | yes | no | no |
| none | | none | | none | | yes | no | no |
| | | | | | | yes | yes | yes |
| | | | | | | yes | yes | yes |
| | | | | | | yes | yes | yes |
| | | | | | | yes | yes | yes |
| | | | | | | yes | no | yes |
| | | | | | | yes | yes | yes |
| | | | | | | yes | yes | yes |
| | | | | | | yes | no | yes |
| | | | | | | yes | yes | yes |
| | | | | | | yes | no | yes |
| none | | none | | none | | yes | no | no |
| none | | none | | none | | yes | no | no |
| None | | None | | None | | yes | no | yes |

| | | | | | | | | |
|---|---|---|---|---|---|---|---|---|
| None | | None | | None | | yes | no | yes |
| None | | None | | None | | yes | no | yes |
| None | | None | | None | | yes | no | yes |
| None | | None | | None | | yes | no | no |
| None | | None | | None | | yes | no | no |
| none | | none | | none | | yes | no | no |
| | | | | | | yes | no | yes |
| | | None | | | | yes | no | yes |
| none | | none | | | | yes | no | yes |
| None | | | | | | yes | yes | yes |
| none | | | | | | yes | no | no |
| | | | | | | yes | yes | yes |
| | | | | | | yes | no | yes |
| None | | | | | | yes | no | no |
| None | | | | | | yes | no | no |
| | | | | | | yes | no | no |
| | | | | | | yes | no | no |
| | | | | | | yes | no | no |
| | | | | | | yes | no | yes |
| | | | | | Good | yes | no | yes |
| | | | | | | yes | no | yes |
| | | | | | | yes | no | yes |
| | | | | | | yes | no | no |
| | | | | | | yes | no | yes |
| | | | | | | yes | no | yes |
| | | | | None | Good | yes | no | no |
| | | | | | | yes | no | no |
| | | | | | | yes | no | yes |
| | | | | | | yes | no | yes |
| | | | | | | yes | yes | yes |
| | | | | | | yes | no | yes |
| | | | | | | yes | no | yes |
| | | | | | | yes | no | yes |
| | | | | | | yes | yes | yes |
| none | | | | | | yes | no | no |
| | | | | | | no | yes | yes |
| | | | | Nil | | yes | yes | yes |
| | | | | | | yes | yes | yes |
| none | | none | | none | | yes | no | yes |
| none | | | | | | yes | no | no |
| | | | | | | yes | no | yes |
| | | | Good | | | yes | no | no |
| | | | | | | yes | no | yes |
| | | | | | | yes | no | yes |
| | | | | | | yes | no | yes |

| | | | | | | | | |
|---|---|---|---|---|---|---|---|---|
| | | | | | | yes | no | no |
| | | | | | | yes | yes | yes |
| | | | | | | yes | no | yes |
| | | | | | | yes | yes | yes |
| we rather do not have every drug . | | | Average | | | yes | no | yes |
| | | | | | | yes | no | yes |
| very hard to even come by medications so patients are usually requeste | | | | | | yes | no | no |
| | | | | | | yes | no | no |
| | | | | | | yes | no | no |
| | | | | | | yes | no | no |
| | | | | | | yes | no | no |
| | | | | | | yes | yes | yes |
| | | | | | | yes | no | yes |
| | | | | | | yes | no | no |
| | | | | | | yes | no | no |
| | | | | | | yes | no | yes |
| | | | | | | yes | no | yes |
| No | | | | | | yes | no | yes |
| | | | | | | yes | no | yes |
| | | | | | | yes | no | no |
| | | | | | | yes | no | yes |
| | | | | | | yes | yes | yes |
| | | | | | | yes | no | no |
| | | | | | | yes | no | no |
| | | | | | | yes | yes | yes |
| | | | | | | yes | no | yes |
| | | | | | | yes | no | no |
| | | | | | | yes | no | no |
| | | | | | | yes | no | yes |
| | | | | | | yes | no | yes |
| | | | | | | yes | yes | yes |
| no | | no | | | | yes | no | no |

| What diabe | What diabe | What diabe | What diabe | What diabe | What diabe | What diabe | Availability | Availability |
|---|---|---|---|---|---|---|---|---|
| no | no | no | no | yes | yes | no | no | yes |
| no | no | yes | no | yes | yes | no | no | no |
| no | no | yes | no | yes | yes | no | yes | yes |
| yes | no | no | no | yes | yes | no | no | no |
| yes | no | no | no | yes | yes | no | no | no |
| no | yes | no | yes | yes | yes | no | yes | no |
| yes | no | yes | yes | yes | yes | no | no | no |
| yes | no | no | no | yes | yes | no | yes | no |
| no | no | no | no | yes | no | no | yes | yes |
| no | no | no | yes | yes | yes | no | yes | no |
| yes | no | yes | no | yes | yes | no | no | no |
| yes | no | yes | yes | yes | yes | no | yes | yes |
| no | no | no | no | yes | no | no | no | no |
| no | no | yes | no | no | no | no | no | no |
| no | no | yes | no | no | no | no | no | no |
| no | no | yes | no | no | no | no | no | no |
| no | no | yes | yes | yes | yes | no | no | no |
| no | no | yes | no | no | no | no | no | no |
| no | no | yes | yes | no | no | no | no | no |
| no | no | yes | no | no | no | no | yes | yes |
| no | no | yes | no | no | no | no | yes | no |
| yes | no | yes | no | no | no | no | no | no |
| no | no | yes | no | no | yes | no | no | no |
| no | no | yes | yes | yes | yes | no | no | no |
| no | no | yes | yes | yes | yes | no | no | no |
| no | no | yes | no | no | no | no | no | no |
| yes | yes | yes | no | no | yes | no | no | no |
| no | no | yes | no | no | no | no | no | no |
| no | no | yes | no | no | no | no | no | no |
| no | no | yes | no | no | no | no | no | no |
| no | no | yes | no | no | no | no | no | no |
| no | no | yes | no | no | no | no | no | yes |
| no | no | yes | yes | yes | yes | no | no | yes |
| yes | no | yes | no | no | no | no | no | no |
| no | no | yes | no | no | no | no | yes | no |
| no | no | yes | no | no | no | no | no | no |
| yes | no | yes | no | no | no | no | no | no |
| yes | no | yes | no | no | no | no | no | no |
| no | no | yes | no | no | no | no | yes | no |
| yes | no | yes | no | no | no | no | no | no |
| no | no | yes | no | no | no | no | no | no |
| no | no | yes | no | no | no | no | no | no |
| no | no | yes | no | no | no | no | no | no |
| no | no | yes | no | no | no | no | no | no |
| no | no | yes | no | no | no | no | no | no |

| | | | | | | | | |
|---|---|---|---|---|---|---|---|---|
| no | no | yes | no | no | no | no | no | no |
| no | no | yes | no | no | no | no | no | no |
| no | no | yes | no | no | no | no | no | no |
| no | no | yes | no | no | no | no | no | no |
| no | no | yes | no | no | no | no | no | no |
| no | no | yes | no | no | no | no | no | no |
| no | no | yes | no | no | no | no | no | no |
| no | no | yes | no | no | no | no | no | no |
| no | no | yes | no | no | no | no | no | no |
| no | no | yes | no | no | no | no | no | no |
| no | no | yes | no | no | no | no | no | no |
| yes | no | yes | no | no | yes | no | no | no |
| no | no | yes | no | no | no | no | no | no |
| no | no | yes | no | no | no | no | no | no |
| no | no | yes | no | no | no | no | no | no |
| no | no | yes | no | no | no | no | no | no |
| no | no | yes | no | no | no | no | no | no |
| no | no | yes | no | no | no | no | no | no |
| no | no | yes | no | no | no | no | no | yes |
| no | no | yes | no | no | no | no | no | no |
| no | no | yes | no | no | no | no | no | no |
| no | no | yes | no | no | no | no | no | no |
| no | no | yes | no | no | no | no | no | no |
| no | no | yes | no | no | no | no | no | no |
| no | no | yes | no | no | no | no | no | no |
| no | no | yes | no | no | no | no | no | no |
| no | no | yes | no | no | no | no | no | no |
| no | no | yes | no | no | no | no | no | no |
| no | no | yes | no | no | no | no | yes | yes |
| no | yes | yes | no | yes | no | no | no | yes |
| no | no | no | no | yes | no | no | yes | yes |
| no | no | yes | no | no | no | no | no | no |
| no | no | yes | no | no | yes | no | no | no |
| no | no | yes | no | yes | yes | no | yes | yes |
| no | no | yes | no | no | no | no | no | no |
| no | no | no | yes | yes | yes | no | no | no |
| no | no | yes | yes | yes | yes | no | no | no |
| no | no | yes | no | yes | yes | no | no | no |
| no | no | yes | no | no | no | no | no | yes |
| no | no | yes | no | no | no | no | no | no |
| no | no | yes | no | no | no | no | no | no |
| no | no | yes | no | no | no | no | no | no |
| no | no | yes | no | no | no | no | no | no |
| no | no | yes | no | no | no | no | yes | no |
| no | no | yes | no | no | no | no | no | no |

| | | | | | | | | |
|---|---|---|---|---|---|---|---|---|
| no | no | yes | no | no | no | no | no | no |
| yes | no | yes | yes | no | yes | no | no | no |
| no | no | yes | no | no | no | no | no | no |
| no | no | yes | no | no | no | no | no | no |
| no | no | yes | no | no | no | no | no | no |
| no | no | yes | no | yes | yes | no | no | no |
| no | no | yes | no | no | no | no | no | no |
| no | no | yes | no | no | no | no | no | no |
| no | no | yes | no | no | no | no | no | no |
| no | no | yes | no | no | no | no | no | no |
| no | no | yes | no | no | no | no | no | no |
| no | no | yes | no | no | no | no | yes | yes |
| no | no | yes | no | no | no | no | no | no |
| no | no | yes | no | no | no | no | no | no |
| no | no | yes | no | no | no | no | no | no |
| no | no | yes | no | yes | no | no | no | no |
| no | no | yes | no | no | no | no | no | no |
| no | no | yes | no | no | no | no | no | no |
| no | no | yes | no | no | yes | no | no | no |
| no | no | yes | no | no | no | no | no | no |
| no | no | yes | no | no | yes | no | no | no |
| yes | no | yes | no | yes | yes | no | no | no |
| no | no | yes | no | no | no | no | no | no |
| no | no | yes | no | no | no | no | no | yes |
| no | no | yes | yes | yes | yes | no | no | no |
| no | no | yes | no | no | no | no | no | no |
| no | no | yes | no | no | no | no | no | no |
| no | no | yes | no | no | no | no | no | no |
| no | no | yes | no | no | no | no | no | no |
| no | no | yes | no | no | no | no | no | no |
| no | no | yes | no | no | no | no | no | no |
| no | no | yes | no | no | no | no | no | no |
