# Supplementary material for: A national audit of facilities, human and material resources for the comprehensive management of diabetes in Ghana-A 2023 update
Source: PLoS One. 2024 May 20;19(5):e0303624. doi: 10.1371/journal.pone.0303624 (PMC11104593; doi:10.1371/journal.pone.0303624)
Supplement: S2 File — (DOCX) [file pone.0303624.s002.docx]

DIABETES CARE IN GHANA:

IDENTIFYING THE GAPS AND UNMET

NEEDS FOR COMPREHENSIVE CARE FORM I: AUDIT OF FACILITIES

Dear Doctor/ Specialist/Administrator,

This study aims at updating the human and material resources as well as identifying the gaps and unmet needs needed for comprehensive diabetes care in Ghana. We would require you to spend up to about 5-10 minutes in providing the information needed. These may range from availability of healthcare personnel, laboratory services, equipment, medications, and healthcare financing available to you in your facility. Kindly provide responses to the following questions as much possible and as candid as you can.

You are to understand that taking part in the research is entirely voluntary. You are further to note that you may refuse to take part or withdraw from the study at anytime without any objection.

Please be assured that no blood or urine sample as well as body measures will be done in this study.

Information we will collect on you in this study will be kept confidential and secure. The information will only be available to the doctors and scientists conducting this study. You are further assured that if a report of this study is prepared for the scientific and medical community you will not be identified by name.

Is there something you do not understand or do you have any questions or concerns about this research? Should you later wish to have any matter or question relating to this study, please do not hesitate to contact any of the research staff personally or alternately call the telephone number provided.

Any queries or concerns should be addressed to the principal investigator, Dr Ernest Yorke at the Diabetes Centre, Korle-Bu, or contact him on phone number (0206301107) or email (pavlovium@yahoo.com) for prompt response.

*Required

1. Email address *

CONSENT

1. By ticking the “Agree” button, I have understood what is going to be done and that I agree to take part in the study. Also, all my concerns have been fully addressed. *

*Mark only one oval.*

Agree

Disagree

TYPE OF HEALTH FACILITY

1. Type of Health facility *

Other:

*Tick all that apply.*

Teaching Hospital

Regional Hospital

Municipal Hospital

District Hospital

1. What region is the health facility located in? *

*Mark only one oval.*

Greater Accra Region

Central Region

Volta Region

Eastern Region

Western Region

Upper East region

Upper West Region

Ashanti Region

Northern Region

West North Region

Bono Region

Ahafo Region

Oti Region

Bono East Region

Savannah Region

North East Region

1. Kindly indicate the name of the district, municipal or metropolitan area the health facility is located in *

AVAILABILITY OF DEDICATED DIABETES CENTRE OR CARE

1. Availability of a Dedicated Diabetes Centre/ Care *

*Mark only one oval.*

yes no

AVAILABILITY OF HEALTH PERSONNEL

# AVAILABILITY OF HEALTH PERSONNEL

Respondents are to tick 'yes' or' no' depending on whether they have the personnel listed in the questionnaire in their facility. Respondents who choose 'yes' are required to indicate the number of personnels they have available.

1. Trained Diabetes Doctors/Specialists * *Mark only one oval.*

yes *Skip to question 6* no *Skip to question 7*

1. If yes , how many are they? *
2. Trained diabetes nurses * *Mark only one oval.*

yes *Skip to question 8* no *Skip to question 9*

1. If yes, how many are they? *
2. Diabetes educators * *Mark only one oval.*

yes *Skip to question 10* no *Skip to question 11*

1. If yes, how many are they? *

Untitled Section

1. Ophthalmic nurses * *Mark only one oval.*

yes *Skip to question 12* no *Skip to question 13*

1. If yes, how many are they? *

Untitled Section

1. Psychologists *

*Mark only one oval.*

yes *Skip to question 14* no *Skip to question 15*

1. If yes, how many are they? *
2. Opthalmologist(s) * *Mark only one oval.*

yes *Skip to question 16* no *Skip to question 17*

1. If yes, how many are they? *
2. Podiatrists/ foot care specialist * *Mark only one oval.*

yes *Skip to question 18*  no *Skip to question 19*

1. If yes, how many are they ? *
2. Foot surgeon(s) / Vascular surgeon(s) *

*Mark only one oval.*

yes *Skip to question 20* no *Skip to question 21*

1. If yes, how many are they ? *
2. Dietician(s) *

*Mark only one oval.*

yes *Skip to question 22* no *Skip to question 23*

1. If yes, how many are they ?

AVAILABILITY OF EQUIPMENT

# AVAILABILITY OF EQUIPMENT

Respondents are to choose' yes' or' no' depending on the availability of stated equipment in their facility. Respondents who choose 'yes' are to indicate the number of the equipment they have available.

1. Sphygmomanometers *

*Mark only one oval.*

yes *Skip to question 24* no *Skip to question 25*

Untitled Section

1. If yes, how many are they ? *
2. Glucometers *

*Mark only one oval.*

yes *Skip to question 26* no *Skip to question 27*

1. If yes, how many are they ? *
2. Ophthalmoscopes *

*Mark only one oval.*

yes *Skip to question 28* no *Skip to question 29*

1. If yes, how many are they ? *
2. Monofilament *

*Mark only one oval.*

yes *Skip to question 30* no *Skip to question 31*

1. If yes, how many are they ? *
2. Biothensiometer *

*Mark only one oval.*

yes *Skip to question 32* no *Skip to question 33*

1. If yes, how many are they ? *
2. Weighing scales *

*Mark only one oval.*

yes *Skip to question 34* no *Skip to question 35*

1. If yes, how many are they ? *

LABORATORY SERVICES AVAILABLE

1. Do you have the following laboratory services available? *

*Mark only one oval per row.*

MEDICATIONS

The following questions seek to assess the availability and supply of diabetes

medications at the pharmacy/dispensary in your facility or

pharmacies/dispensaries nearby for patients to acquire?

yes

no

Full blood count

Urine dipstix for microalbumin

Urine dipstix for proteinuria

Laboratory Microalbumin Estimation

Glycated Haemoglobin

Lipids

Renal function test

Insulin autoantibodies

C-peptide estimation

Liver function Test

# INSULIN- HUMAN INSULIN

1. Availability of Soluble insulin *

*Mark only one oval.*

yes *Skip to question 37* no *Skip to question 38*

SUPPLY OF SOLUBLE INSULIN

1. If yes kindly indicate the supply of Soluble insulin? *

*Mark only one oval per row.*

Below average

Average

Good

Excellent

Supply

AVAILABILTY OF NPH

1. Availability of NPH *

*Mark only one oval.*

yes *Skip to question 39* no *Skip to question 40*

SUPPLY OF NPH

1. If yes, kindly indicate the supply of NPH? *

*Mark only one oval per row.*

Below average

Average

Good

Excellent

Supply

AVAILABILITY OF PREMIXED HUMAN INSULIN

1. Availability of Premixed human Insulin *

*Mark only one oval.*

yes *Skip to question 41* no *Skip to question 42*

SUPPLY OF PREMIXED HUMAN INSULIN

1. If yes kindly indicate the supply of Premixed human Insulin *

*Mark only one oval per row.*

Below average

Average

Good

Excellent

Supply

# INSULIN -ANALOGUE INSULIN

1. Availability of Fast-acting analogue Insulin (Novolog/Novorapid (aspart), Apidra

(glulisine), Humalog (lispro), etc * *Mark only one oval.*

yes *Skip to question 43* no Skip to question 44

SUPPLY OF FAST ACTING ANALOGUE INSULIN

1. If yes, kindly indicate the supply of fast-acting analogue Insulin *

*Mark only one oval per row.*

Below average

Average

Good

Excellent

Supply

AVAILABILITY OF INTERMEDIATE ACTING ANALOGUE INSULIN

1. Availability of Intermediate-acting analogue Insulin *

*Mark only one oval.*

yes *Skip to question 45* no *Skip to question 46*

SUPPLY OF INTERMEDIATE ACTING ANALOGUE INSULIN

1. If yes, kindly indicate the supply of Intermediate acting analogue Insulin *

*Mark only one oval per row.*

Below average

Average

Good

Excellent

Supply

AVAILABILITY OF PREMIXED ANALOGUE INSULIN (e.g Humalog Mix 30, Humalog, NovoMix 30)

1. Availability of Premixed analogue insulin *

*Mark only one oval.*

yes *Skip to question 47* no *Skip to question 48*

SUPPLY OF PREMIXED ANALOGUE INSULIN

1. If yes, kindly indicate the supply of Premixed analogue insulin *

*Mark only one oval per row.*

Below average

Average

Good

Excellent

Supply

AVAILABILITY OF LONG-ACTING INSULIN (e.g Lantus, Levemir)

1. Availability of Long-acting Insulin *

*Mark only one oval.*

yes *Skip to question 49* no *Skip to question 50*

SUPPLY OF LONG-ACTING INSULIN

1. If yes, kindly indicate the supply of Long-Acting Insulin *

*Mark only one oval per row.*

Below average

Average

Good

Excellent

Supply

AVAILABILITY OF ULTRA LONG-ACTING INSULIN(DEGLUDEC/TRESIBA)

1. Availability of Ultra Long acting Insulin (Degludec/Tresiba) *

*Mark only one oval.*

yes *Skip to question 51* no *Skip to question 52*

SUPPLY OF ULTRA LONG-ACTING INSULIN(DEGLUDEC/TRESIBA)

1. If yes, kindly indicate the supply of Ultra Long-Acting Insulin (Degludec/Tresiba) *

*Mark only one oval per row.*

Below average

Average

Good

Excellent

Supply

# OTHER MEDICATIONS

1. Availability of Metformin *

*Mark only one oval.*

yes *Skip to question 53* no *Skip to question 54*

SUPPLY OF METFORMIN

1. If yes, kindly indicate the supply of Metformin *

*Mark only one oval per row.*

Below average

Average

Good

Excellent

Supply

AVAILABILITY OF SULPHONYLUREA (e.g glibenclamide (daonil), glimepiride, gliclazide etc)

1. Availability of Sulphonylurea *

*Mark only one oval.*

yes *Skip to question 55* no *Skip to question 56*

SUPPLY OF SULPHONYLUREA

1. If yes, kindly indicate the supply of Sulphonylurea *

*Mark only one oval per row.*

Below average

Average

Good

Excellent

Supply

AVAILABILITY OF DPP IV INHIBITORS(e.g Januvia (Sitagliptin), Galvus (Vildagliptin, Onglyza (Saxagliptin, Tradjenta (Linagliptin)

1. Availability of DPP IV Inhibitors *

*Mark only one oval.*

yes *Skip to question 57* no *Skip to question 58*

SUPPLY OF DPP IV INHIBITORS

1. If yes, kindly indicate the supply of DPP IV Inhibitors *

*Mark only one oval per row.*

Below average

Average

Good

Excellent

Supply

AVAILABILITY OF SGLT2 INHIBITORS(e.g dapagliflozin (Forxiga), empagliflozin

(Jardiance), ertugliflozin (Steglatro), empagliflozin (Jardiance)

1. Availability of SGLT 2 Inhibitors *

*Mark only one oval.*

yes *Skip to question 59* no Skip to question 60

SUPPLY OF SGLT 2 INHIBITORS

1. If yes, kindly indicate the supply of SGLT 2 Inhibitors *

*Mark only one oval per row.*

Below average

Average

Good

Excellent

Supply

AVAILABILITY OF THIAZOLIDINEDIONES(e.g. Pioglitazone, Rosiglitazone)

1. Availability of Thiazolidinediones *

*Mark only one oval.*

yes *Skip to question 61* no *Skip to question 62*

SUPPLY OF THIAZOLIDINEDIONES

1. If yes, kindly indicate the supply of Thiazolidinediones *

*Mark only one oval per row.*

Below average

Average

Good

Excellent

Supply

AVAILABILITY OF GLP-1 ANALOGUES (e.g Exenatide (Bydureon/ Byetta),

Semaglutide (Ozempic/ Rybelsus), Dulaglutide (Trulicity) Liraglutide (Victoza), Lixisenatide (Adlyxin

1. Availability of GLP-1 analogues *

*Mark only one oval.*

yes *Skip to question 63* no *Skip to question 64*

SUPPLY OF GLP-1 ANALOGUES

1. If yes, kindly indicate the supply of GLP-1 analogues *

*Mark only one oval per row.*

Below average

Average

Good

Excellent

Supply

Other

medications

available in your

facility that are

not stated in

the

questionnaire

Respondents are to respond to the availability and supply of the other

medications not listed in the preceding question. Where Response 1 is the

first medication listed, Response 2 is the second medication listed and

Response 3 is the third medication listed below.

1. Other medications available in your facility that are not stated in the questionnaire

SUPPLY OF RESPONSE 1

1. If yes, kindly indicate the supply of Response 1

*Mark only one oval per row.*

Below average

Average

Good

Excellent

Supply

SUPPLY OF RESPONSE 2

1. If yes, kindly indicate the supply of Response 2

*Mark only one oval per row.*

Below average

Average

Good

Excellent

Supply

SUPPLY OF RESPONSE 3

1. If yes, kindly indicate the supply of Response 3

*Mark only one oval per row.*

Below average

Average

Good

Excellent

Supply

PAYMENT SCHEME

Tick as many as apply

1. Payment Scheme *

*Mark only one oval per row.*

yes

no

NHIS

Private Insurance

Cash and Carry

GUIDELINES AND PROTOCOLS

1. What diabetes guidelines do you use at your facility? *

*Mark only one oval per row.*

yes

no

Local

Regional

National Treatment Guidelines

National, Other

International

Combination

None

AVAILABILITY OF DIABETES ASSOCIATION & DIABETES SUPPORT GROUPS AT YOUR FACILITY OR CATCHMENT AREA

1. Availability of *

*Mark only one oval per row.*

yes

no

Diabetes Association

Diabetes Support Groups

This content is neither created nor endorsed by Google.

[Forms](https://www.google.com/forms/about/?utm_source=product&utm_medium=forms_logo&utm_campaign=forms)
