# Supplementary material for: A national audit of facilities, human and material resources for the comprehensive management of diabetes in Ghana-A 2023 update
Source: PLoS One. 2024 May 20;19(5):e0303624. doi: 10.1371/journal.pone.0303624 (PMC11104593; doi:10.1371/journal.pone.0303624)
Supplement: Attachment — Submitted filename: PONE-D-23-31002_Editor.pdf [file pone.0303624.s003.pdf]

PLOS ONE

A national audit of facilities, human and material resources for the comprehensive management of diabetes in Ghana-A 2023 update

--Manuscript Draft--

**Manuscript Number:**	PONE-D-23-31002
**Article Type:**	Research Article
**Full Title:**	A national audit of facilities, human and material resources for the comprehensive management of diabetes in Ghana-A 2023 update
**Short Title:**	A national audit of facilities and resources for the comprehensive management of diabetes
**Corresponding Author:**	Ernest Yorke<br>University of Ghana Medical School<br>Accra, GHANA
**Keywords:**	Audit, Comprehensive Diabetes Care, Diabetes, Resources, Survey, Unmet needs.
**Abstract:**	Introduction<br>The human and material resources as well as the systems for managing diabetes in Africa are inadequate. This study or needs assessment, aimed at updating the human and material resources, identifying the gaps and unmet needs for comprehensive diabetes care in Ghana.<br>Methods: We conducted a national audit of 122 facilities in all 16 administrative regions of Ghana. Information obtained covered areas on personnel and multidisciplinary teams, access to medications, access to laboratory services, financing, screening services, management of diabetes complications, and availability/use of diabetes guidelines or protocols. Data was analysed using STATA version 16.1. P-values <0.05 were set as significant.<br>Results:<br>Only 85(69.7%) out of the 122 surveyed facilities had a dedicated centre or service for diabetes care. Twenty-eight (23%) had trained diabetes doctors/specialists; and whilst most centres had ophthalmic nurses and dieticians, majority of them did not have trained diabetes educators (nurses), psychologists, ophthalmologists, podiatrists, and foot/vascular surgeons. Also, 13.9% had monofilaments, none could perform urine dipstick for microalbumin; 5 (4.1%) and just over 50% could perform laboratory microalbumin estimation and glycated haemoglobin respectively. Access to and supply of human insulins was better than analogue insulin in most centres. Nearly 100% of the institutions surveyed had access to metformin and sulphonylurea with good to excellent supply in most cases, whilst access to Sodium Glucose Transporter-2 inhibitors and Glucagon-like peptide-1 analogues were low, and moderate for Dipeptidyl peptidase-4 inhibitors and thiazolidinediones.<br>Overwhelming majority of the health facilities (95.1%) offered NHIS as payment mechanism for clients, whilst 68.0% and 30.3% of the patients paid for services using out-of-pocket and private insurance respectively. Fifteen facilities (12.3%) had Diabetes Support Groups in their locality and catchment areas.<br>Conclusion: Significant gaps remain in human and material resources, which requires urgent multisectoral collaboration to promote and achieve acceptable comprehensive diabetes care.
**Order of Authors:**	Ernest Yorke
	Josephine Akpalu
	Gwendolyn de-Graft Johnson
	Yacoba Atiase
	Margaret Reynolds
	Ruth Laryea
	John Tetteh
	Alfred E. Yawson

Powered by Editorial Manager® and ProduXion Manager® from Aries Systems Corporation

	Albert A.G. Amoah
**Opposed Reviewers:**	
**Additional Information:**	
**Question**	**Response**
**Financial Disclosure**<br><br>Enter a financial disclosure statement that describes the sources of funding for the work included in this submission. Review the submission guidelines for detailed requirements. View published research articles from *PLOS ONE* for specific examples.<br><br>This statement is required for submission and **will appear in the published article** if the submission is accepted. Please make sure it is accurate.<br><br>**Funded studies**<br>Enter a statement with the following details:<br>• Initials of the authors who received each award<br>• Grant numbers awarded to each author<br>• The full name of each funder<br>• URL of each funder website<br>• Did the sponsors or funders play any role in the study design, data collection and analysis, decision to publish, or preparation of the manuscript?<br><br>Did you receive funding for this work?	Yes
Please add funding details.<br>as follow-up to "**Financial Disclosure**<br><br>Enter a financial disclosure statement that describes the sources of funding for the work included in this submission. Review the submission guidelines for detailed requirements. View published research articles from *PLOS ONE* for specific examples.<br><br>This statement is required for submission and **will appear in the published article** if the submission is accepted. Please make	This study funded jointly by the authors and a mid-career grant from the University of Ghana (UG-ORID 13th Call: MCG-002/12-20) to EY, JA, YA and AEY. The funder did not play any role in the study design, data collection and analysis, decision to publish, or preparation of the manuscript.<br>Sponsor URL is https://orid.ug.edu.gh/

Powered by Editorial Manager® and ProduXion Manager® from Aries Systems Corporation

sure it is accurate.<br><br>**Funded studies**<br>Enter a statement with the following details:<br>• Initials of the authors who received each award<br>• Grant numbers awarded to each author<br>• The full name of each funder<br>• URL of each funder website<br>• Did the sponsors or funders play any role in the study design, data collection and analysis, decision to publish, or preparation of the manuscript?<br><br>Did you receive funding for this work?"	
Please select the country of your main research funder (please select carefully as in some cases this is used in fee calculation).<br>as follow-up to "**Financial Disclosure**<br><br>Enter a financial disclosure statement that describes the sources of funding for the work included in this submission. Review the submission guidelines for detailed requirements. View published research articles from *PLOS ONE* for specific examples.<br><br>This statement is required for submission and **will appear in the published article** if the submission is accepted. Please make sure it is accurate.<br><br>**Funded studies**<br>Enter a statement with the following details:<br>• Initials of the authors who received each award<br>• Grant numbers awarded to each author<br>• The full name of each funder<br>• URL of each funder website<br>• Did the sponsors or funders play any role in the study design, data collection and analysis, decision to publish, or preparation of the manuscript?<br><br>Did you receive funding for this work?"	GHANA - GH

**Competing Interests**<br><br>Use the instructions below to enter a competing interest statement for this submission. On behalf of all authors, disclose any competing interests that could be perceived to bias this work—acknowledging all financial support and any other relevant financial or non-financial competing interests.<br><br>This statement is required for submission and **will appear in the published article** if the submission is accepted. Please make sure it is accurate and that any funding sources listed in your Funding Information later in the submission form are also declared in your Financial Disclosure statement.<br><br>View published research articles from *PLOS ONE* for specific examples.<br><br>**NO authors have competing interests**<br><br>Enter: *The authors have declared that no competing interests exist.*<br><br>**Authors with competing interests**<br><br>Enter competing interest details beginning with this statement:<br><br>*I have read the journal's policy and the authors of this manuscript have the following competing interests: [insert competing interests here]*<br><br>* typeset	The authors have declared that no competing interests exist
**Ethics Statement**<br><br>Enter an ethics statement for this submission. This statement is required if the study involved:<br><br>• Human participants<br>• Human specimens or tissue<br>• Vertebrate animals or cephalopods	All participants provided written informed consent. Ethical and Protocol approval for the study was sought from the University of Ghana College of Health Sciences Ethical and Protocol Review Committee with reference number CHS-Et/M2 5.15/2019-2020. It complied with the Helsinki Declaration of 1964 (Revised 2013) on human experimentation. Strict confidentiality of data and privacy for study participants were ensured. Data was kept secured and was available only to the principal investigator.

• Vertebrate embryos or tissues<br>• Field research<br><br>Write "N/A" if the submission does not require an ethics statement.<br><br>General guidance is provided below. Consult the submission guidelines for detailed instructions. **Make sure that all information entered here is included in the Methods section of the manuscript.**	

Powered by Editorial Manager® and ProduXion Manager® from Aries Systems Corporation

**Format for specific study types**<br><br>**Human Subject Research (involving human participants and/or tissue)**<br>• Give the name of the institutional review board or ethics committee that approved the study<br>• Include the approval number and/or a statement indicating approval of this research<br>• Indicate the form of consent obtained (written/oral) or the reason that consent was not obtained (e.g. the data were analyzed anonymously)<br><br>**Animal Research (involving vertebrate animals, embryos or tissues)**<br>• Provide the name of the Institutional Animal Care and Use Committee (IACUC) or other relevant ethics board that reviewed the study protocol, and indicate whether they approved this research or granted a formal waiver of ethical approval<br>• Include an approval number if one was obtained<br>• If the study involved *non-human primates*, add *additional details* about animal welfare and steps taken to ameliorate suffering<br>• If anesthesia, euthanasia, or any kind of animal sacrifice is part of the study, include briefly which substances and/or methods were applied<br><br>**Field Research**<br><br>Include the following details if this study involves the collection of plant, animal, or other materials from a natural setting:<br>• Field permit number<br>• Name of the institution or relevant body that granted permission	
**Data Availability**<br><br>Authors are required to make all data underlying the findings described fully available, without restriction, and from the time of publication. PLOS allows rare exceptions to address legal and ethical concerns. See the PLOS Data Policy and FAQ for detailed information.	Yes - all data are fully available without restriction

Powered by Editorial Manager® and ProduXion Manager® from Aries Systems Corporation

A Data Availability Statement describing where the data can be found is required at submission. Your answers to this question constitute the Data Availability Statement and **will be published in the article**, if accepted.<br><br>**Important:** Stating 'data available on request from the author' is not sufficient. If your data are only available upon request, select 'No' for the first question and explain your exceptional situation in the text box.<br><br>Do the authors confirm that all data underlying the findings described in their manuscript are fully available without restriction?	
**Describe where the data may be found in full sentences. If you are copying our sample text, replace any instances of XXX with the appropriate details.**<br><br>• If the data are **held or will be held in a public repository**, include URLs, accession numbers or DOIs. If this information will only be available after acceptance, indicate this by ticking the box below. For example: *All XXX files are available from the XXX database (accession number(s) XXX, XXX.*).<br>• If the data are all contained **within the manuscript and/or Supporting Information files**, enter the following: *All relevant data are within the manuscript and its Supporting Information files.*<br>• If neither of these applies but you are able to provide **details of access elsewhere**, with or without limitations, please do so. For example:<br><br>*Data cannot be shared publicly because of [XXX]. Data are available from the XXX Institutional Data Access / Ethics Committee (contact via XXX) for researchers who meet the criteria for access to confidential data.*<br><br>*The data underlying the results presented in the study are available from (include the name of the third party*	All relevant data are within the manuscript and its Supporting Information files.

and contact information or URL).<br>• This text is appropriate if the data are owned by a third party and authors do not have permission to share the data.<br><br>* typeset	
Additional data availability information:	

Powered by Editorial Manager® and ProduXion Manager® from Aries Systems Corporation

17/09/23

The Editor
PLOS ONE

Dear Editor,

A National Audit of Facilities, Human and Material Resources for the Comprehensive Management of Diabetes Mellitus in Ghana-A 2023 Update

Sub-Saharan African countries is expected to experience the largest increases in the prevalence of diabetes by the year 2045, which will be driven mainly by type 2 diabetes. Diabetes related complications are also expected to increase. However, the human and material resources as well as the systems for managing diabetes in Africa are inadequate.

We conducted a national audit of 122 facilities in all 16 administrative regions of Ghana. Information obtained covered areas on personnel and multidisciplinary teams, access to medications, access to laboratory services, financing, screening services, management of diabetes complications, and availability/use of diabetes guidelines or protocols.

The study found out that Only 85(69.7%) out of the 122 surveyed facilities had a dedicated centre or service for diabetes care. Twenty-eight (23%) had trained diabetes doctors/specialists; and whilst most centres had ophthalmic nurses and dieticians, majority of them did not have trained diabetes educators (nurses), psychologists, ophthalmologists, podiatrists, and foot/vascular surgeons. Access to and supply of human insulins was better than analogue insulin in most centres. Nearly 100% of the institutions surveyed had access to metformin and sulphonylurea with good to excellent supply in most cases, whilst access to Sodium Glucose Transporter-2 inhibitors and Glucagon-like peptide-1 analogues were low, and moderate for Dipeptidyl peptidase-4 inhibitors and thiazolidinediones.

About 30% of the patients paid for services using out-of-pocket Also, 91.8% surveyed used the National Treatment Guideline in their practice and 15 (12.3%) had Diabetes Support Groups in their locality and catchment areas.

These findings suggest that significant gaps remain in human and material resources, which requires urgent multisectoral collaboration to promote and achieve acceptable comprehensive diabetes care.

We pray that you publish this manuscript to bring the findings to the fore to engender change in policy to reverse this trend in Ghana.

Yours sincerely,

SIGNED

Dr Ernest Yorke

Corresponding Author

Manuscript

Click here to access/download;Manuscript;Audit of Facilities_PLOS One.docx

Title

A national audit of facilities, human and material resources for the comprehensive management of diabetes in Ghana-A 2023 update

Short Title: A national audit of facilities and resources for the comprehensive management of diabetes

Authors

Ernest Yorke[1*], Josephine Akpalu[1], Gwendolyn de-Graft Johnson[1], Yacoba Atiase[1], Margaret Reynolds[1], Ruth Laryea[1], John Tetteh[2], Alfred E. Yawson[2] and Albert G. B Amoah[1]

1. *Department of Medicine & Therapeutics, University of Ghana Medical School, College of Health Sciences, P.O. Box GP 4236, Accra, Ghana*
2. *Department of Community Health, University of Ghana Medical School, College of Health Sciences, P.O. Box GP 4236, Accra, Ghana*

**Corresponding Author Address: Dr Ernest Yorke, Department of Medicine & Therapeutics, University of Ghana Medical School, College of Health Sciences, P.O. Box GP 4236, Accra, Ghana*

Corresponding Author Email: eyorke@ug.edu.gh

ABSTRACT

Introduction

The human and material resources as well as the systems for managing diabetes in Africa are inadequate. This study or needs assessment, aimed at updating the human and material resources, identifying the gaps and unmet needs for comprehensive diabetes care in Ghana.

Methods: We conducted a national audit of 122 facilities in all 16 administrative regions of Ghana. Information obtained covered areas on personnel and multidisciplinary teams, access to medications, access to laboratory services, financing, screening services, management of diabetes complications, and availability/use of diabetes guidelines or protocols. Data was analysed using STATA version 16.1. *P-values* <0.05 were set as significant.

Results:

Only 85(69.7%) out of the 122 surveyed facilities had a dedicated centre or service for diabetes care. Twenty-eight (23%) had trained diabetes doctors/specialists; and whilst most centres had ophthalmic nurses and dieticians, majority of them did not have trained diabetes educators (nurses), psychologists, ophthalmologists, podiatrists, and foot/vascular surgeons. Also, 13.9% had monofilaments, none could perform urine dipstick for microalbumin; 5 (4.1%) and just over 50% could perform laboratory microalbumin estimation and glycated haemoglobin respectively. Access to and supply of human insulins was better than analogue insulin in most centres. Nearly 100% of the institutions surveyed had access to metformin and sulphonylurea with good to excellent supply in most cases, whilst access to Sodium Glucose Transporter-2 inhibitors and Glucagon-like peptide-1 analogues were low, and moderate for Dipeptidyl peptidase-4 inhibitors and thiazolidinediones.

Overwhelming majority of the health facilities (95.1%) offered NHIS as payment mechanism for clients, whilst 68.0% and 30.3% of the patients paid for services using out-of-pocket and

private insurance respectively. Fifteen facilities (12.3%) had Diabetes Support Groups in their locality and catchment areas.

Conclusion: Significant gaps remain in human and material resources, which requires urgent multisectoral collaboration to promote and achieve acceptable comprehensive diabetes care.

Keywords: *Audit, Comprehensive Diabetes Care, Diabetes, Resources, Survey, Unmet needs.*

INTRODUCTION

The incidence and prevalence of especially type 2 diabetes are increasing due to sedentary lifestyles, obesity, and poor dietary habits. This would bring with it both microvascular (neuropathy, retinopathy and nephropathy) and macrovascular (stroke, myocardial infarction and peripheral vascular disease) complications [1]. The doctor-patient ratio in Ghana has been improving steadily over the past few years with ratio in 2016 estimated to be about 1: 8,481 [2]. However, this is still below the recommended ratio of 1:1,000 by the World Health Organisation (WHO) [3]. Doctors with specialist knowledge or interest in diabetes in Ghana are conservatively put at 30, a situation that is woefully inadequate to achieve optimum care.

In 1998, A study by Amoah *et al* revealed that only Korle-Bu Teaching Hospital (KBTH) had diabetologists among the 5 regional hospitals surveyed [4]. Dedicated centres for managing diabetes were also inadequate with only one functional diabetes clinic in KBTH. Currently, Ghana can boast of 5 public teaching hospitals, 10 regional hospitals, over 100 district and municipal hospitals, hundreds of health centres and over a 6000 small community service centres referred to as National Community Health Planning and Services (CHPS) compounds or zones [5]. Out of these, many do not have specialised centres for diabetes care. Most diabetes patients are therefore treated as part of the general clinic and

hospital care. The study by Amoah *et al* [4] also revealed inadequacies in other trained staff, equipment and laboratory support services. Specifically, only 2 facilities had dieticians, 2 facilities had eye specialists, no facility had trained diabetes educators, there was no chiropodist or podiatrist in any of the facilities, clinical care equipment were lacking except for sphygmomanometers. Laboratory tests for managing diabetes were also found to be inadequate. Biochemical assays were largely available; however, glycated haemoglobin estimation could only be done at one centre, and no centre offered c-peptide, insulin autoantibodies and urine microalbumin estimation. Whilst the supply of oral diabetes medications was relatively acceptable at the time, insulin supply was erratic. No facility had chronic haemodialysis service. At the time there was neither a diabetes advisory board nor guidelines for managing diabetes in Ghana at all levels in Ghana. Data on diabetes morbidity and mortality were scanty and there were 3 diabetes associations nationwide.

Financing diabetes care is a challenge because of the high cost involved and this imposes a lot of stress on patients with diabetes and chronic diseases in general [1, 6]. In 2015, a study of 40 households found out the extended family was the main resource of financial support for diabetes treatment and management [7]. The National Health Insurance Scheme (NHIS) established in 2006 has only partially addressed the financing gap in diabetes care [7]. Whilst, metformin, sulphonylureas, thiazolidindiones and human insulins are covered under the NHIS, relatively newer medications like dipeptidyl peptidase IV (DPP IV) inhibitors, glucagon-like peptide1-1 (GLP-1) analogues, sodium glucose transporter 2 (SGLT 2) inhibitors and analogue insulins are not covered under the scheme [8]. The benefit package of NHIS tariffs also does not include cost of glucose monitoring meters and related devices, podiatry services, some aspects of eye care including laser therapy for diabetic retinopathy among others. Late reimbursement of costs to health facilities deny them of the much-needed resources, which also affects quality of care [8, 9]. At the present time, there are no national or regional guidelines.

for the management of diabetes

An assessment of the current state of diabetes care was urgently needed to identify the gaps and possible solutions for improvement. A system which is robust in terms of personnel, physical facilities, diabetes care equipment, adequate and affordable medicines and laboratory services, financing and governmental support is needed to address current and future challenges.

This study or needs assessment sought to update the human and material resources as well as identify the gaps and unmet needs for comprehensive diabetes care in Ghana since the last update some 25 years ago.

MATERIALS AND METHODS

Study design and settings

This national audit was a cross-sectional study conducted from May 2021 to October 2021. It involved a total of 122 facilities in all 16 administrative regions of Ghana comprising 6 teaching (including 37 Military Hospital), 10 regional, 100 municipal/district hospitals, and 6 private/quasi-government hospitals.

Recruitment, information gathering and ethical considerations

The Ghana Health Service (GHS) through the Non-communicable Disease Directorate provided an introductory letter granting the lead investigators and research assistants permission to undertake the survey in public health facilities, and to facilitate their entry and acceptance in these facilities. All public Teaching Hospitals, Regional Hospitals, most Municipal and District Hospitals in Ghana were contacted for the survey. The 37-Military Hospital Accra, a level-4 United Nations Hospital (and which also operates essentially as a Teaching Hospital) in Accra as well as some private and quasi-government health facilities

were purposively selected. There were two teams for the nationwide assessment, one covering the southern half of the country, and the other the northern half of the country.

Respondents were consented hospital administrators, medical directors and superintendents, and healthcare professionals involved in diabetes care in these facilities. A pretested questionnaire was used for data capturing and either administered in-person or by telephone. Information obtained centered on their current state of diabetes care in terms of facilities and resources as well as existing gaps in managing diabetes. Specific areas assessed included personnel, multidisciplinary teams, access to medications, access to laboratory services, financing, screening services, management of diabetes complications, and availability/use of diabetes guidelines or protocols as well as support groups.

All participants provided written informed consent. Ethical and Protocol approval for the study and all the sites involved was sought from the University of Ghana College of Health Sciences Ethical and Protocol Review Committee with reference number CHS-Et/M2 5.15/2019-2020. It complied with the Helsinki Declaration of 1964 (Revised 2013) on human experimentation.

Data handling and Statistical Methods

Data handling codes were assigned to respondents for confidentiality. Only the principal investigator matched codes to respondents. Questionnaires were stored in a safety cabinet under lock and key whilst collected data were entered and stored into an electronic database which was password protected. Statistical analysis of the data were entered into Microsoft Excel version 12 and imported into the STATA version 16.1 for analysis. After this assessment, data were analysed according to the thematic arrears outlined in the data extraction tools. Descriptive statistics of the responses were summarized in appropriate frequency tables and flow charts. Categorical variables

were summarized as frequencies and percentages whilst continuous variables were summarized as means. Responses were examined for internal consistencies. Significance level was set at a p-value less than 0.05.

RESULTS

In all, one-hundred and twenty-two out of a total of 150 health facilities contacted responded, giving a response rate of 81.3% (***Figure 1: Recruitment Algorithm***). This comprised of 6 teaching (4.9%), 10 regional (8.2%), 100 district/municipal (82%) and 6 private/quasi-government (4.9 %) hospitals and health facilities. All the 16 administrative regions of Ghana were represented with Greater Accra, Eastern and Ashanti regions contributing 16.2% (n=19), 15.4% (n=18) and 12.8% (n=15) respectively (Table1).

Table 1: Type of Health Facilities and Geographical Locations

**Type of health facility**	**Frequency (n=122)**	**%**
Teaching Hospital	6	5.7
Regional Hospital	10	4.1
District/Municipal Hospital	100	86.1
Private/Quasi-Government	6	4.1
**Region**		
Ahafo	9	7.7
Ashanti	15	12.8
Bono East	3	2.6
Bono	7	6.0
Central	9	7.7
Eastern	18	15.4
Greater Accra	19	16.2
North-East	2	1.7
Northern	3	2.6
Oti	1	0.9
Savannah	2	1.7
Upper East	10	8.5
Upper West	7	6.0
Volta	4	3.4
Western North	6	5.1
Western	2	1.7

Only 85(69.7%) out of the 122 surveyed facilities had a dedicated centre or service for diabetes care (Table 2), with 6 (7.0%), 10 (11.8%) and 4 (4.7%) being from the teaching, regional and private/quasi-government health facilities respectively. Twenty-eight (23%) had trained diabetes doctors/specialists with a total of 9 (32.1%) from the teaching and regional hospitals. Whilst most centres had ophthalmic nurses (87.7%) and dieticians (64%), majority of them did not have trained diabetes educators (nurses) (69.7%), psychologists (95%), ophthalmologists (74.6%), podiatrists/ foot care specialists (95.1%) and foot/vascular surgeons (92.6%).

Table 2: Availability of Diabetes Care Centres and Multi-Disciplinary Care Team

**Variable**	**Response**		**Min-Max**	**Mean±SD**	**Median(IQR)**
	**No n(%)**	**Yes n(%)**			
Availability of a Dedicated Diabetes Centre/ Care	37(30.3)	85(69.7)	NA		
Trained Diabetes Doctors/Specialists	94(77.0)	28(23.0)	1-8	2.78±2.17	5(1-4)
Diabetes educators	85(69.7)	37(30.3)	1-4	1.9±1.11	1.5(1-3)
Ophthalmic nurses	15(12.3)	107(87.7)	1-6	2.28±1.08	2(2-3)
Psychologists	95(77.9)	27(22.1)	1-15	2.1±0.65	2(2-4)
Ophthalmologist	91(74.6)	31(25.4)	1-17	2.2±0.65	2(2-4)
Podiatrists/ foot care specialists	116(95.1)	6(4.9)	1-2	1.4±0.55	1(1-2)
Foot surgeon(s) / Vascular surgeon(s)	113(92.6)	9(7.4)	1-4	1.7±1.09	1(1-2)
Dietician(s)	58(47.5)	64(52.5)	1-20	2.1±0.67	3(2-4)

Min: minimum; Max: maximum; SD: standard deviation; IQR: inter quartile range

Table 3: Equipment and Laboratory Services Available for Diabetes Care

**Equipment**	**Absent n(%)**	**Present n(%)**
Sphygmomanometers	1(0.8)	121(99.2)
Glucometers	7(5.7)	115(94.3)

Ophthalmoscopes	14(11.5)	108(88.5)
Monofilament	105(86.1)	17(13.9)
Biothesiometer	120(98.4)	2(1.6)
Weighing scales	2(1.6)	120(98.4)
**Laboratory Services Available**		
Full blood count	9(7.4)	113(92.6)
Urine dipstick for microalbumin	122 (100)	0 (0.0)
Urine dipstick for proteinuria	8(6.6)	114(93.4)
Laboratory microalbumin estimation	117 (95.9)	5 (4.1)
Glycated Haemoglobin	54(44.3)	68(55.7)
Lipids	32(26.2)	90(73.8)
Renal function test	34(27.9)	88(72.1)
Insulin autoantibodies	117 (95.9)	5 (4.1)
C-peptide estimation	117 (95.9)	5 (4.1)
Liver function Test	35(28.7)	87(71.3)

Whilst majority of facilities (about 90% or more) has sphygmomanometers, glucose monitoring devices, weighing scales and ophthalmoscopes, only 1.6% and 13.9% had biothesiometers and monofilaments respectively (Table 3). None of the institutions could perform urine dipstick for microalbumin, and only 5 (4.1%) could perform laboratory microalbumin estimation. Just over 50% could perform the very important glycated haemoglobin whilst only 5 (4.1%) could perform insulin autoantibodies and c-peptide estimation. Majority (>70%) were able to perform lipid and renal function tests (Table 3).

The majority (>80%) of the health facilities had access to soluble and premix human insulins with a good to excellent supply in most instances. Only 36.9% had access to or use NPH insulin. Access to fast-acting analogue insulin, premixed analogue, long-acting and ultra-long-acting insulins were recorded in 24.6%, 30.3%, 35.2% and 15.6% out of the 122 institutions surveyed (Table 4). Nearly 100% of the institutions surveyed had access to metformin and sulphonylurea with good to excellent supply in most cases, whilst access to SGLT-2 inhibitors

and GLP-1 analogues were low, (17.2% and 9.8% respectively); and 23.0% and 38.0 % of the health facilities had access to DPP-4 inhibitors and thiazolidinediones respectively (Table 4).

Table 4: Medication Availability and Supply to the Health Facilities

**Medication Availability**	**Frequency n(%)**	**Supply**			
		**Below average n(%)**	**Average n(%)**	**Good n(%)**	**Excellent n(%)**
**Soluble Insulin**					
no	3(2.5)				
yes	119(97.5)	7 (5.9)	21(17.7)	65(54.6)	26(21.8)
**NPH**					
no	77(63.1)				
yes	45(36.9)	2(4.4)	10(22.2)	26(57.8)	7(15.6)
**Premixed human Insulin**					
no	19(15.6)	3(2.9)	20(19.4)	64(62.1)	16(15.5)
yes	103(84.4)				
**Fast-acting analogue Insulin**					
no	92(75.4)				
yes	30(24.6)	5(16.7)	7(23.3)	15(50.0)	3(10.0)
**Premixed analogue insulin**					
no	85(69.7)				
yes	37(30.3)	3(8.1)	9(24.3)	18(48.6)	7(18.9)
**Long-acting Insulin**					
**no**	79(64.8)				
**yes**	43(35.2)	6(14.0)	8(18.6)	19(44.2)	10(23.3)
**Ultra Long-acting Insulin (Degludec/Tresiba)**					
no	103(84.4)				
yes	19(15.6)	5(26.3)	4(21.1)	7(36.8)	3(15.8)
**Metformin**					
no	1(0.8)				

yes	121(99.2)	1(0.8)	9(7.4)	67(55.4)	44(36.4)
**Sulphonylurea**					
no	3(2.5)				
yes	119(97.5)	2(1.7)	8(6.7)	71(59.7)	38(31.9)
**DPP-4 Inhibitors**					
no	94(77.0)				
yes	28(23.0)	4(14.3)	8(28.6)	11(39.3)	5(17.9)
**SGLT-2 Inhibitors**					
no	101(82.8)				
yes	21(17.2)	4(19.0)	3(14.3)	10(47.6)	4(19.0)
**Thiazolidinediones**					
no	84(68.9)				
yes	38(31.1)	5(13.2)	9(23.7)	14(36.8)	10(26.3)
**GLP-1 analogues**					
no	110(90.2)				
yes	12(9.8)	5(41.7)	3(25.0)	4(33.3)	

NPH: Neutral Protamine Hagedorn; SGLT-2: Sodium-Glucose Cotranporter-2; DPP-4: Dipeptidyl Peptidase 4; GLP-1: Glucagon-like Peptide-1

Table 5: Payment Schemes, Guidelines and Protocols and Support Groups

Variable	No n(%)	Yes n(%)
**Payment Scheme**		
NHIS	6(4.9)	116(95.1)
Private Insurance	85(69.7)	37(30.3)
Cash and Carry	39(32.0)	83(68.0)
**Guidelines and Protocols**		
Local	107(87.7)	15(12.3)
Regional	119(97.5)	3(2.5)
National Treatment Guidelines	10(8.2)	112(91.8)
National, other	109(89.3)	13(10.7)
International	95(77.9)	27(22.1)
Combination	93(76.2)	29(23.8)
**Diabetes Association & Diabetes Support Groups (at Facility or Catchment Area)**		
Diabetes Association	110(90.2)	12(9.8)
Diabetes Support Groups	107(87.7)	15(12.3)

NHIS: National Health Insurance Scheme

Overwhelming majority of the health facilities (95.1%) offered NHIS as payment mechanism for clients, whilst 68.0% and 30.3% of the patients paid for services using cash-and-carry and private insurance respectively, according to the facilities surveyed. One-hundred and twelve out of the 122 facilities (91.8%) surveyed used the National Treatment Guidelines (NTG) in their practice whilst 12.3% used a combination of either NTG, local, regional, or international guidelines (Table 5). Only 15 (12.3%) and 12 (9.8%) of the health facilities had Diabetes Support Groups and Associations respectively in their locality and catchment areas.

DISCUSSION

This study seeks to update the audit done 25 years go by Amoah *et al [4]*, which characterised facilities and resources for diabetes care in regional hospitals in southern Ghana. It found inadequate dedicated centres for diabetes care, limited laboratory tests, equipment, and poor supply of medications especially insulin. In many ways, this study is more comprehensive: it covered all 10 existing regional hospitals, most district and municipal hospitals, all teaching hospitals including 37 military hospital, and some selected private and quasi-government hospitals.

Only 85(69.7%) out of the 122 surveyed facilities had a dedicated centre or service for diabetes care (Table 2), with 6 (7.0%), 10 (11.8%) and 4 (4.7%) being from the teaching, regional and private/quasi-government health facilities respectively. This suggests that 65 (65%) out of the 100 districts and municipal hospitals had a dedicated centre or service for diabetes care. Whilst this is an improvement, a lot more effort needs to be made to establish such centres in these districts to improve access to diabetes care. In the interim the Ghana Health Service Wellness Clinics concept [10] could be strengthened to provide diabetes care services to patients.

It was noteworthy that most institutions that were surveyed had dieticians and ophthalmic nurses, a situation that would enhance non-pharmacological control of diabetes and screening for eye complications respectively. The availability of trained diabetes nurses/educators,

psychologists, ophthalmologists remain a challenge in most centres except the teaching and regional hospitals. Very few centres had podiatrists/ foot care specialists and foot/vascular surgeons. [illegible]oot care is an important aspect of diabetes care in preventing and managing diabetes-related foot problems. New strategies such as curriculum development, training and funding are urgently needed to produce and deploy more footcare specialists, as well as foot and vascular surgeons.

Whilst most centres had basic equipment like sphygmomanometers, glucose monitoring devices, weighing scales and ophthalmoscopes, only 1.6% and 13.9% had biothesiometers and monofilaments respectively. The absence of the latter two makes the detection and subsequent management of diabetes neuropathy difficult. The study found out that majority of the health facilities surveyed could perform basic biochemical tests such as lipid and renal function tests, none of the institutions could perform urine dipstick for microalbumin, and only 5 (4.1%) could perform laboratory microalbumin estimation. It was surprising to find out that just over 50% could perform the very important glycated haemoglobin, which is need for diagnosis in some instances and crucially for monitoring [11]. This situation must improve, and in the least, with the provision of affordable point-of-care devices for the measurement of HbA1c. Healthcare professionals must be educated on the need to periodically request HbA1c measurement to ascertain the level of glucose control in their patients. They must also engage the managers of such institutions to procure such equipment and devices.

Access to and supply of human insulins, metformin and sulphonylurea was good in most facilities, whilst that for analogue insulins, SGLT-2 inhibitors and GLP-1 analogues were low. Although, more expensive, the latter three offers some advantages in terms of less risk of hypoglycaemia and weigh gain (analogue insulins), weight loss and cardiovascular benefits (SGLT-2 inhibitors and GLP-1 analogues) [12] [13]. Price reduction strategies such as waiver

of import taxes and duties could help in making these relatively newer medications more accessible to patients.

Overwhelming majority of the health facilities (95.1%) offered NHIS as payment mechanism for clients, whilst 68.0% and 30.3% of the patients paid for services using cash-and-carry (out-of-pocket) and private insurance respectively. Financing diabetes care is a challenge because of the high cost involved, and this imposes a lot of stress on sufferers of diabetes and chronic diseases in general [1, 6]. In 2015, a study of 40 households found out the extended family was the main resource of financial support for diabetes treatment and management [7]. Whilst access to NHIS as a payment mechanism has improved since its establishment in 2006, it has only partially addressed the financing gap in diabetes care [9]. Whilst human insulins and older oral antiglycaemic agents such as metformin and sulphonylurea are covered by NHIS, relatively newer ones like dipeptidyl peptidase-4 (DPP-4) inhibitors, glucagon-like peptide-1 (GLP-1) analogues, sodium glucose transporter 2 (SGLT 2) inhibitors and analogue insulins are not covered by under the scheme [8]. The benefit package of NHIS tariffs also does not include cost of glucose monitoring meters and related devices, many relevant laboratory tests (such as glycated haemoglobin and urine microalbumin estimation). Podiatry services, some aspects of eye care including laser therapy for diabetic retinopathy among others are also not covered by the NHIS. Other problems that have bedevilled the NHIS scheme are unrealistic tariffs for services as well as late reimbursement of costs to health facilities, which denies them of the much-needed resources, which also affects quality of care [8, 9].

One-hundred and twelve out of the 122 facilities (91.8%) surveyed used the National Treatment Guidelines (NTG) in their practice whilst 12.3% used a combination of either NTG, local, regional, or international guidelines. Since the audit in 1998 [4], Ghana still does not have a

comprehensive national guidelines for diabetes care. The ongoing effort by the Ministry of Health, GHS and partners like the Diabetes Endocrine & Metabolic Society of Ghana (DEMSoG) to develop one must be expedited. In the absence o[illegible]pecialist or trained personnel in diabetes, guidelines help to decentralize diabetes best practices and across the country.

Only 15 (12.3%) and 12 (9.8%) of the health facilities had Diabetes Support Groups [illegible] Associations respectively in their locality and catchment areas. Diabetes support group and associations provide patient support, counselling, advocacy and sometimes free or affordable consumables and medications such as glucose monitoring devices and insulin. Their formation and must be encouraged and supported to grow in many localities across the country.

CONCLUSIONS

This study sought to update the audit done 25 years ago by Amoah *et al* [4], which characterised facilities and resources for diabetes care in regional hospitals in southern Ghana. Whilst a lot of improvement in human and material resources were noted, significant gaps remain, which requires urgent multisectoral collaboration to promote and achieve acceptable comprehensive diabetes care in Ghana.

ACKNOWLEDGEMENTS

We thank the Diabetes Endocrine and Metabolic Society of Ghana (DEMSoG) for their endorsement of the project.

References

1. Sun H, Saeedi P, Karuranga S, Pinkepank M, Ogurtsova K, Duncan BB, et al: IDF Diabetes Atlas: Global, regional and country-level diabetes prevalence estimates for 2021 and projections for 2045. Diabetes Res. Clin. Pract. 2022, 183:109119.

2. Nsiah-Asare A: The Health Sector in Ghana: Facts and Figures 2017. Accra: Ghana Health Service 2017. Available at https://www.moh.gov.gh/wp-content/uploads/2017/07/Facts-and-figures-2015.pdf. Accessed 21/06/23.

3. Organization WH: World Health Statistics 2010: World Health Organization; 2010.Available https://www.who.int/publications-detail-redirect/9789241563987. Accessed 21/06/23.

4. Amoah AG, Owusu SK, Saunders JT, Fang WL, Asare HA, Pastors JG, et al: Facilities and resources for diabetes care at regional health facilities in southern Ghana. Diabetes Res. Clin. Pract. 1998, 42(2):123-130.

5. The Health Sector In Ghana Facts And Figures, 2018. Available at https://africaopendata.org/dataset/the-health-sector-in-ghana-facts-and-figures-2018. Accessed 21/06/23.

6. Aikins Ad-G, Agyei-Mensah S, Agyemang C: Chronic non-communicable diseases in Ghana. Multidisciplinary Perspectives 2014, 1.

7. Pei F: Managing diabetes in urban Ghana: is it affordable? : Duke University; 2015. Available at https://dukespace.lib.duke.edu/dspace/bitstream/handle/10161/10014/Pei_duke_0066N_12921.pdf;sequence=1. Accessed 21/06/23.

8. National Health Insurance Scheme: Benefit Package. Available at https://www.nhis.gov.gh/benefits. Accessed 21/06/23.

9. Fusheini A, Marnoch G, Gray AM: Implementation challenges of the National Health Insurance Scheme in selected districts in Ghana: evidence from the field. Int. J. Public Adm. 2017, 40(5):416-426.

10. Hospital GAR: Family Medicine and Wellness Centre. https://garhgovgh/services/family-medicine-wellness-services/ 2017. Accessed 21/06/23.

11. World Health Organisation: Definition and diagnosis of diabetes mellitus and intermediate hyperglycaemia: report of a WHO/IDF consultation. 2006. Available at https://www.who.int/publications-detail-redirect/definition-and-diagnosis-of-diabetes-mellitus-and-intermediate-hyperglycaemia. Accessed 21/06/23.

12. Lopaschuk GD, Verma S: Mechanisms of cardiovascular benefits of sodium glucose co-transporter 2 (SGLT2) inhibitors: a state-of-the-art review. JACC: Basic Transl. Sci. 2020, 5(6):632-644.

13. Boyle JG, Livingstone R, Petrie JR: Cardiovascular benefits of GLP-1 agonists in type 2 diabetes: a comparative review. Clinical Science 2018, 132(15):1699-1709.

Figure
Click here to access/download;Figure;Figure 1.docx

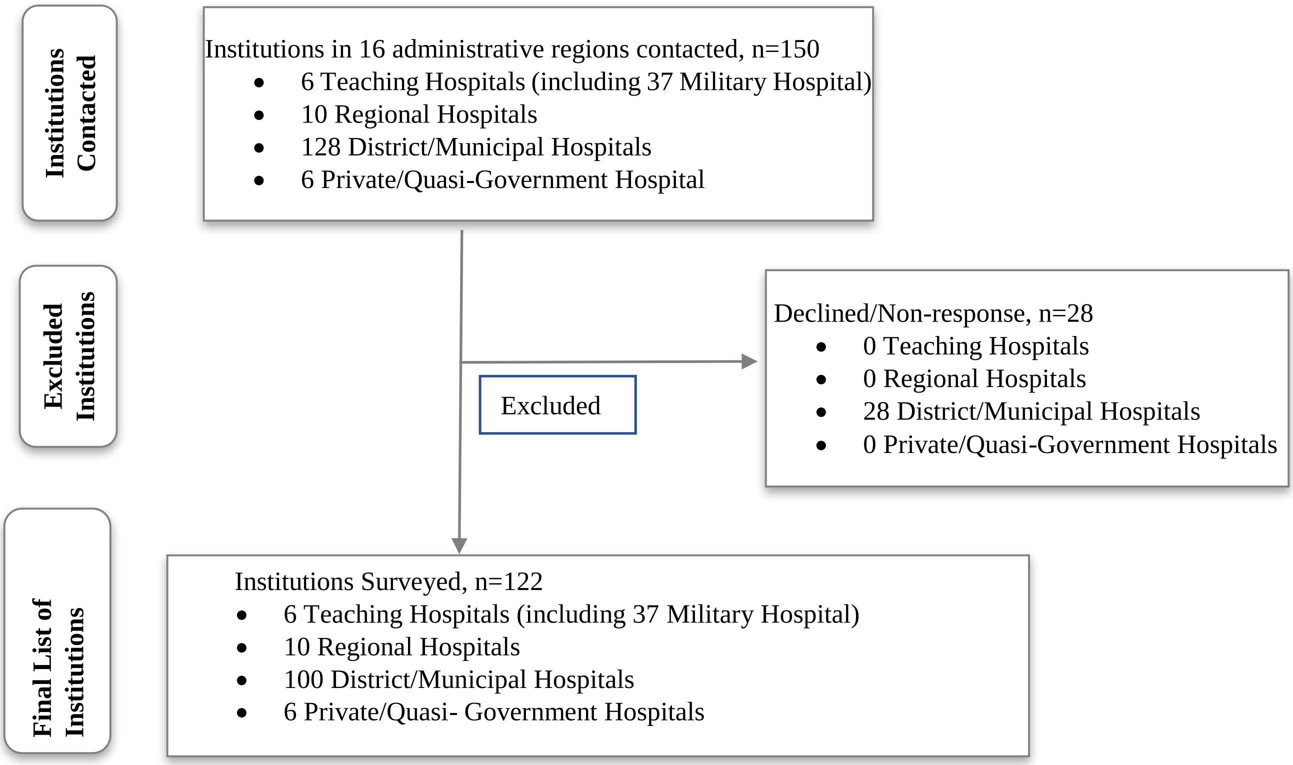

Figure 1

Click here to access/download
Other
PLOSOne_Human_Subjects_Research_Checklist.docx

Click here to access/download
Other
ETHICAL CLEARANCE_ext.pdf

Click here to access/download
Other
Ethical Clearance_diabetes care.pdf
