# Supplementary material for: A national audit of facilities, human and material resources for the comprehensive management of diabetes in Ghana-A 2023 update
Source: PLoS One. 2024 May 20;19(5):e0303624. doi: 10.1371/journal.pone.0303624 (PMC11104593; doi:10.1371/journal.pone.0303624)
Supplement: Attachment — Submitted filename: Responses to Reviewers.docx [file pone.0303624.s004.docx]

**Responses to Reviewers**

| **No.** | **Comment** | **Response** | **Page no.** |
| --- | --- | --- | --- |
| 1. | The authors must add a section on the prevalence of T2D in Ghana. | This suggestion has been effected. The new sentences now read as:  *Global prevalence of diabetes among adults aged 20-79 years in 2021 was estimated by the International Diabetes Federation (IDF) to be 537 million, which is predicted to rise to 783 million by 2045, with the largest increases expected in sub-Saharan African countries [1]. Estimated figures by IDF for Ghana were 329,200 in 2021, representing about 2% of the adult population [1]. However, community-based studies suggest a much higher prevalence than stated [2].*  *The rising incidence and prevalence of especially type 2 diabetes are due to sedentary lifestyles, obesity, and poor dietary habits [3]. This would bring with it both microvascular (neuropathy, retinopathy and nephropathy) and macrovascular (stroke, myocardial infarction and peripheral vascular disease) complications [1, 3]. The expected increases in diabetes-related morbidity and mortality require corresponding improvements in human and material resources.* | 3 |
| 2. | Provide reference for the statement “The incidence and prevalence of especially type 2 diabetes are increasing due to sedentary lifestyles, obesity, and poor dietary habits | This suggestion has been effected. The new sentences now read as:  *The rising incidence and prevalence of especially type 2 diabetes are due to sedentary lifestyles, obesity, and poor dietary habits [3].* | 3 |
| 3. | Kindly situate this work within a global conversation on diabetes burden | This suggestion has been effected. The new sentences now read as:  *Global prevalence of diabetes among adults aged 20-79 years in 2021 was estimated by the International Diabetes Federation (IDF) to be 537 million, which is predicted to rise to 783 million by 2045, with the largest increases expected in sub-Saharan African countries [1]. Estimated figures by IDF for Ghana were 329,200 in 2021, representing about 2% of the adult population [1]. However, community-based studies suggest a much higher prevalence than stated [2].*  *The rising incidence and prevalence of especially type 2 diabetes are due to sedentary lifestyles, obesity, and poor dietary habits [3]. This would bring with it both microvascular (neuropathy, retinopathy and nephropathy) and macrovascular (stroke, myocardial infarction and peripheral vascular disease) complications [1, 3]. The expected increases in diabetes-related morbidity and mortality require corresponding improvements in human and material resources.* | 3 |
| 4. | include refs  https://journals.plos.org/plosone/article?id=10.1371/journal.pone.0194677  Even community interventions are also low: https://pubmed.ncbi.nlm.nih.gov/36398761/ | The suggested reference has been added as reference no. 6  The suggested reference has been added as reference no. 7 | 3  4 |
| 5. | The rationale is very weak; to pick from one study conducted in the 1990s.  Please read these  https://journals.sagepub.com/doi/10.1177/13558196221111708#:~:text=Diabetes%20clinics%20for%20outpatients%20are,health%20insurance%20scheme%2C%20the%20NHIS. | These reference and inferences have been incorporated as part of the rationale under the introduction as references 10 | 4 |
| 6 | Take note of this reference: https://www.medrxiv.org/content/10.1101/2023.04.19.23288806v1.full | The suggested reference and inference have been added as reference no. 12 |  |
| 7. | ref  Is T2D treatment approach not provided in the standard treatment guidelines 2017 | Whilst it is true that some aspects of the Standard Treatment Guidelines, it is not comprehensive enough. The amended sentence now reads:  *“Whilst Standard Treatment Guidelines for Ghana has portions on the management of diabetes, it is not comprehensive and is also restricted for use at the primary and secondary levels. A truly national and comprehensive guidelines for the management of diabetes dedicated to all levels of care was unavailable at the time of this survey.”* | 5 |
| 8. | Add the last 2 paragraphs of the ‘Introduction’ | This suggestion has been effected to now read:  *“An assessment of the current state of diabetes care was urgently needed to identify the gaps and possible solutions for improvement. A system which is robust in terms of personnel, physical facilities, diabetes care equipment, adequate and affordable medicines and laboratory services, financing and governmental support is needed to address current and future challenges. This study or needs assessment sought to update the human and material resources as well as identify the gaps and unmet needs for comprehensive diabetes care in Ghana since the last update some 25 years ago.”* | 5 |
| 9. | were the team trained. please include how long and the content of the training | Yes, the team was trained. The now included statement reads:  *“They were trained over 3-day period in community/facility entry protocols, selection of responsible respondents, questionnaire administration, data capture and entry.”* | 6 |
| 10. | add the questionnaire as a supplementary file | This has been added. |  |
| 11. | Add a space: | The suggestion on line 202 has been effected in the sentence:  *“Only 85 (69.7%) out of the 122 surveyed facilities had a dedicated centre…….”* | 8 |
| 12 | in your methods include a section on study variables.  Write about the kinds of professionals needed for diabetes and provide refs. and indicate that you asked about these in your study | A subsection on study variables now reads:  *“Study variables: Information obtained centered on their current state of diabetes care in terms of facilities and resources as well as existing gaps in managing diabetes. Specific areas assessed included personnel, multidisciplinary teams (trained diabetes doctors/specialists, diabetes educators and nurses, ophthalmic nurses, psychologists, ophthalmologists, foot care specialists/podiatrists dieticians and foot or vascular surgeons) [16], access to medications, access to laboratory services, financing, screening services, management of diabetes complications, and availability/use of diabetes guidelines or protocols as well as support groups.”* | 6,7 |
| 13. | please introduce all these variables in the methods section | This effected in comment no. 12 above | 6,7 |
| 14 | a cross tabulation between these variables and the type of facility will be appropriate | This suggestion has been effected in tables 2,3 and 4 | 9,10,13 |
| 15. | be consistent with spacing (results) | This suggested has been complied with throughout the results section |  |
| 16. | use upper case  Please design your tables well. and professionally using excel. | The tables have been redesigned . They are clearer in the clean document |  |
| 17. | why dont the facilities have the required eduipment. the authors should reflect on this question and revise their discussion accordingly | These possible reasons have been carefully adduced in the discussion section | 14-17 |
|  | the authors repeat most of the results in the discussion, the focus should be on the why | This section has been incorporated in the discussion section | 14-17 |
| 18 | add the study limitations  add the section on the implication of the findings | 2 subsections, study limitations and implications of study findings have now been added. Another sentence has also been added to the conclusion | 18 |
|  |  |  |  |
