# Supplementary material for: A national audit of facilities, human and material resources for the comprehensive management of diabetes in Ghana-A 2023 update
Source: PLoS One. 2024 May 20;19(5):e0303624. doi: 10.1371/journal.pone.0303624 (PMC11104593; doi:10.1371/journal.pone.0303624)
Supplement: Attachment — Submitted filename: PLOS ONE.docx [file pone.0303624.s005.docx]

This study or needs assessment, aimed at updating the human and material resources, identifying the gaps and unmet needs for comprehensive diabetes care in Ghana is well written.

However, there are few questions I have for the authors to answers and comments they have to consider

1. Kindly explained train diabetes doctors, are they diabetologist or medical doctors that have been trained to treat patients with diabetes? How many diabetologist do you have in Ghana?
2. Diabetes educators, what qualifications do they have? (Well trained educators or health care workers trained to educate patients with diabetes)
3. Few typographical errors? Authors should kindly read through the manuscript to correct all grammatical and typographical errors
4. Does the national treatment guideline give comprehensive approach in treating diabetes? What is the target health care workers to use national treatment guideline?
