# Supplementary material for: A national audit of facilities, human and material resources for the comprehensive management of diabetes in Ghana-A 2023 update
Source: PLoS One. 2024 May 20;19(5):e0303624. doi: 10.1371/journal.pone.0303624 (PMC11104593; doi:10.1371/journal.pone.0303624)
Supplement: Attachment — Submitted filename: Responses to Reviewers.docx [file pone.0303624.s007.docx]

**Responses to Reviewers**

| **No.** | **Comment** | **Response** | **Page no.** |
| --- | --- | --- | --- |
| 1. | The discussion repeats a lot of the findings presented in the results. Kindly edit. Present a one paragraph on key findings without stats(if possible) and dedicate the majority of the discussion to explaining the results | Thanks for the suggestion. This suggestion has been effected.  References to the detailed results have been edited out or reduced dramatically in the discussion. These have been done as tack changes. | 15-18 |
| 2. | Kindly explained train diabetes doctors, are they diabetologist or medical doctors that have been trained to treat patients with diabetes? How many diabetologist do you have in Ghana? | Thanks for the comment. Trained diabetes doctors are doctors who have basic medical qualification and have acquired additional training in diabetes care such as short courses and certificates. They are lumped with diabetologists in this study, who are doctors and have acquired additional specialist qualification in diabetes care or endocrinology. Such diabetologists may be internal medicine physicians, endocrinologist, or family physicians.  The number of diabetologists including endocrinologists are approximately 20.  Trained diabetes doctors/diabetologists provide specialised diabetes care to patients. | N/A |
| 3. | Diabetes educators, what qualifications do they have? (Well trained educators or health care workers trained to educate patients with diabetes) | Thanks for the comment.  The term ‘diabetes educators’ as used in this study loosely refers to health care workers who are certified diabetes educators or have received some form of training to provide diabetes education (without certification).  This was done considering the limited number of certified diabetes educators in Ghana. | N/A |
| 4. | Few typographical errors? Authors should kindly read through the manuscript to correct all grammatical and typographical errors | Thanks for the comment.  Grammatical and typographical errors have been corrected throughout the manuscript and highlighted a track changes. |  |
| 5. | Does the national treatment guideline give comprehensive approach in treating diabetes? What is the target health care workers to use national treatment guideline? | Thanks for the comment.  The Standard Treatment Guidelines (STGs) does not give comprehensive approach in treating diabetes. It covers treatment approaches for a wide range of diseases.  Although, the ministry of health suggests that the STGs could be used at all levels of care, in practice, it is used mostly at the primary and secondary levels of care (health centres, district, and regional hospitals). This has been highlighted in the discussion section on page 17. Tertiary level practitioners will mostly rely on other guidelines from bodies such as the American Diabetes Association. | 17-18 |
| 6. | Can you add one possible recommendation at facility level (Abstract conculsion) | Thanks for the comment.  The new conclusion of the Abstract reads: “*An urgent multisectoral collaboration, including prioritisation of resources at the facility level, to promote and achieve acceptable comprehensive diabetes care is required.”* | 3 |
| 7. | Please indicate that diabetes is structured to start at the health centers. CHPS have not been designed to treat NCDs according to their protocols | Thanks for the comment.  The suggestion has been incorporated. The new sentence reads: *“Notwithstanding that CHPS compounds are not designated to treat diabetes, many of the other facilities do not have specialised centres for diabetes care. Most diabetes patients are therefore treated as part of the general clinic and hospital care.”* | 4 |
